# Relationship Between Gut Microbiota and Phenylalanine Levels: A Mendelian Randomization Study

**DOI:** 10.1002/mbo3.70148

**Published:** 2025-11-07

**Authors:** Zhijuan Liu, Minggang Ye, Huiya Jin, Weixia Chen, Hasitiyaer Jieens, Rui Han

**Affiliations:** ^1^ Department of Prenatal Diagnosis, Reproductive Medicine Center The First Affiliated Hospital of Xinjiang Medical University Urumqi China; ^2^ Department of Operation Management The First Affiliated Hospital of Xinjiang Medical University Urumqi China; ^3^ Department of Neonatology, Maternal‐fetal Medicine Center The First Affiliated Hospital of Xinjiang Medical University Urumqi China

**Keywords:** gut microbiota, Mendelian randomization, phenylalanine, phenylketonuria

## Abstract

The specific members of the gut microbiota linked to phenylketonuria remain to be identified. This study aimed to assess the association between gut microbiota on phenylalanine (Phe) levels using a two‐sample Mendelian randomization (MR) approach. Summary statistics from genome‐wide association studies (GWAS) related to individual gut microbiota were obtained from the MiBioGen Global Consortium database. The data set of Phe levels was derived from GWAS summary datasets. Inverse variance weighting (IVW) served as the primary method to infer the causal relationship between gut microbiota and Phe levels. Additional pleiotropy and heterogeneity tests were conducted to evaluate the reliability of the findings. The *Family XIII AD3011* group had a protective effect on Phe levels (OR = 0.962, 95% CI: 0.942–0.982, *p* < 0.001), and these associations remained significant after FDR correction (adjusted *p*‐value = 0.027). There was no evidence of notable heterogeneity and horizontal pleiotropy among the instrumental variables. Our data indicate that *Family XIII AD3011 group* is associated with reduced Phe levels, highlighting a potential link between gut microbiota and Phe levels. Although MR analysis supports a causal relationship, it may not precisely estimate the effect size, necessitating further studies to validate these findings and quantify the association.

## Graphical abstract

This study showed that *Family XIII AD3011 group* was associated with reduced Phe levels, providing important evidence to reveal the interaction between gut microbiota and Phe levels, and providing a scientific basis for the development of personalized diet and nutrition recommendations for PKU patients.

## 1 Introduction

Phenylalanine (Phe) is an essential amino acid vital for protein synthesis and overall metabolic (Church et al. 2020). Its levels are primarily regulated by phenylalanine hydroxylase (PAH), a tetrahydrobiopterin (BH_4_)‐dependent rate‐limiting enzyme that catalyzes the conversion of Phe to tyrosine (Tyr) with BH4 serving as an essential co‐substrate (Hillert et al. 2020). Deficiency in PAH leads to the accumulation of this amino acid in the blood and brain, resulting in diseases such as phenylketonuria (PKU) (Matuszewska et al. 2024). In untreated PKU patients, blood Phe levels are significantly elevated, promoting the formation of phenylketone bodies excreted in the urine (van Spronsen et al. 2021). This eventually leads to developmental delays, severe intellectual disability (with an intelligence quotient of below 30), seizures, severe behavioral difficulties, and psychiatric disorders (van Wegberg et al. 2017; Waisbren et al. 2007). Currently, the management of PKU is highly depending on the Phe‐restricted dietary (Rodrigues et al. 2021). This approach effectively reduces the Phe concentration and its metabolites in the blood and urine, thereby mitigating PKU‐related complications such as intellectual disability, behavioral problems, and neurological symptoms (Rondanelli et al. 2023; Moritz et al. 2023).

The gut microbiota, a diverse community of microorganisms inhabiting in the human gastrointestinal tract (Thursby and Juge 2017), plays a crucial role in regulating physiological processes through the production of various metabolites that influence host phenotype and overall health (TD VAWJSES 2018; Rath and Dorrestein 2012). For example, the gut microbiota is involved in nutrient metabolism, immune modulations, and may influence host behavior (De Palma et al. 2015; Montiel‐Castro et al. 2013). Alterations in the microbiota have been associated with an increased risk of neurological disorders, inflammatory bowel disease, obesity, diabetes, and cardiovascular disease. Its composition and diversity are shaped by various factors, among which diet is particularly important (Perler et al. 2023). Therefore, different dietary patterns lead to distinct characteristic bacterial profiles (Zimmer et al. 2012). In PKU patients, a prescribed diet consisting of a Phe‐free l‐amino acid mixture and specially formulated low‐specific foods helps to limit natural protein intake while ensuring adequate amino acid supply and supporting normal growth (van Spronsen et al. 2017). However, this dietary intervention may also alter the gut microbiota. Studies have indicated significant differences in the gut microbiota of PKU patients compared to healthy individuals (Pinheiro de Oliveira et al. 2016; Su et al. 2021), suggesting a potential role of the gut microbiota in the clinical manifestations and treatment responses of PKU patients. Nevertheless, it remains unclear whether these microbial changes are a consequence of the PKU‐specific diet, or a cause of the disease, or a results of its metabolic alterations.

Only a small number of studies have used the 16S rRNA sequencing to explore the association between gut microbiota and PKU. However, the microbial differences may have been influenced by small cohort sizes and potential confounding variables (Pinheiro de Oliveira et al. 2016; Bassanini et al. 2019). Mendelian randomization (MR) utilizes genetic variants as instrumental variable (IV) to infer and measure associations within observational epidemiological research (Yao et al. 2023). It mitigates several limitations inherent to observational studies through reducing the confounding factors such as age, drug or environmental exposure, and reverse causation (Lawlor et al. 2008). To date, no studies have investigated the potential involvement of gut microbiota in regulating Phe levels using the MR framework. In the present study, we performed a two‐sample MR analysis based on large‐scale genome‐wide association study (GWAS) to evaluate the potential causal link between gut microbiota and Phe levels.

## 2 Materials and Methods

### 2.1 Study Design

**FIGURE 1:**
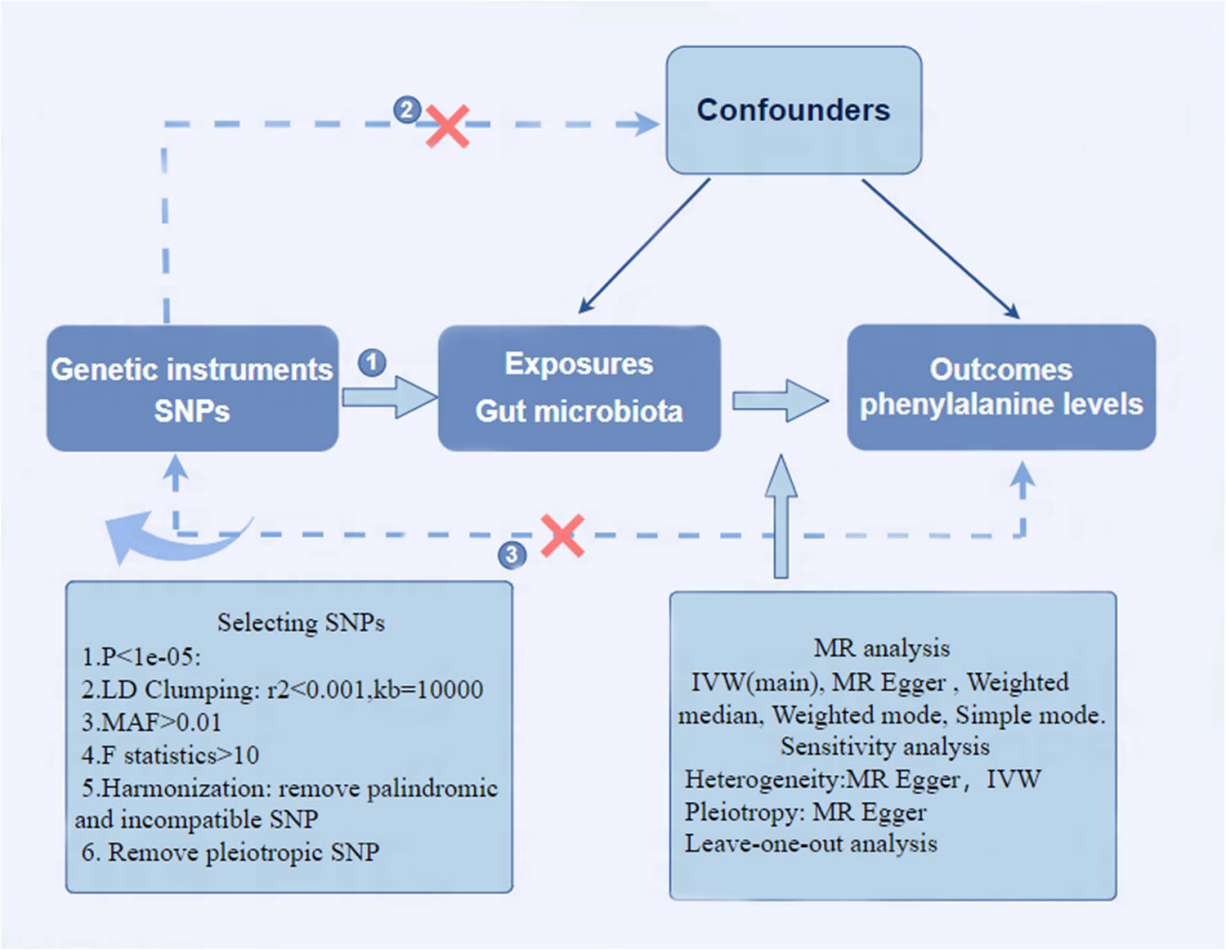
Schematic diagram of the two‐sample Mendelian randomization study design.

We employed a two‐sample MR analysis method to explore the potential relationship between gut microbiota and Phe levels. An overview of the study design is presented in Figure 1. To minimize bias from confounding variables, the MR approach is based on three essential assumptions: (a) Single‐nucleotide polymorphisms (SNPs) strongly linked to gut microbiota are selected as IVs; (b) IVs must be independent of confounding factors; (c) IVs must be independent of the outcome only through exposure, without alternative pathways. As this analysis relies solely on previously published and publicly available GWAS summary statistics, no additional ethical approval was necessary.

### 2.2 Data Sources

This study utilized publicly accessible summary statistics from GWAS. Data on the human gut microbiota were sourced from the largest genome‐wide meta‐analysis of gut microbiota composition published to date by the MiBioGen Consortium (Table S1). The data set comprised 16S rRNA gene sequencing profiles and genotyping data from 18,340 subjects from 24 cohorts (Kurilshikov et al. 2021). To minimize heterogeneity, only data from 130 genera were included. The GWAS IDs are ebi‐a‐GCST90016959 to ebi‐a‐GCST90017088. Additionally, to reduce potential population stratification bias, only participants of European descent were included in the MR analysis.

Genetic variants related to Phe levels were identified using summary data from a GWAS conducted by Richardson TG et al (Richardson et al. 2022), which included 115,030 European samples. Data for this study were accessed from the IEU OpenGWAS project (ID: ebi‐a‐GCST90092936; URL: https://gwas.mrcieu.ac.uk/). Metabolic signatures were derived from blood samples of 115,082 UK Biobank participants, an unprecedented sample size.

### 2.3 IVs Selection

IVs were selected using the following criteria: (a) SNPs linked to each genus at a global significance threshold (*p* < 5.0 × 10^−5^) were considered as potential IVs (Sanna et al. 2019); (b) The European 1000 Genomes Project data set served as the reference panel, to account linkage disequilibrium (LD) between SNPs, retaining only the SNPs with the lowest *p*‐value were retained among the SNPs with *R* ^2^ < 0.001 (aggregation window size = 10,000 kb) (Ming‐Gang Deng et al. 2023); (c) SNPs with minor allele frequency (MAF) ≤ 0.01 were excluded (Li et al. 2022); (d) Strong IVs with *F* > 10 calculated using the following formula:

$$R^{2}=2\times \left(1-\mathrm{MAF}\right)\times \mathrm{MAF}\times {\left(\beta /\right)}^{2},$$

SD=SE×√N,

$$F=R^{2}\times \left(N-2\right)/-(1-R^{2}),$$

where MAF represents the MAF; *β* denotes the effect size of each SNP on the exposure; SD and SE are the standard deviation and standard error of the effect size, respectively, and *N* indicates the sample size of the exposure GWAS data set.

### 2.4 Two‐Sample MR Analysis

All statistical analyses were performed using R version 4.3.2, incorporating the “TwoSampleMR” and “fdrtool” packages. Five approaches were utilized for the analysis, including MR Egger, weighted median, inverse variance weighted (IVW), simple mode, and weighted mode. First, the IVW was used as the primary method to estimate the overall causal effect of gut microbiota on Phe levels by aggregating SNP‐level associations.

Sensitivity analyses were carried out to evaluate the reliability of significant MR findings and to rule out potential bias from heterogeneity and pleiotropy within IVs. MR Egger, Weighted median, Simple mode, and Weighted mode were used for sensitivity analysis. Heterogeneity was assessed using MR‐Egger and IVW, with *p* > 0.05 interpreted as no evidence of heterogeneity. The Egger intercept was used to analyze horizontal pleiotropy, where a *p* > 0.05 was interpreted as evidence against its presence. When the IVW produced a *p* < 0.05 without evidence of horizontal pleiotropy, the result was considered unbiased (Holmes et al. 2017). Furthermore, leave‐one‐out analysis was performed to detect any disproportionately influence of individual SNP on the overall estimate (Sanderson et al. 2022). The false discovery rate (FDR) correction was performed using the Benjamini–Hochberg (BH) method (Chen et al. 2023). In the presence of an adjusted *p* < 0.05, it was indicative of a potential relationship between the intestinal flora genus and Phe levels.

## 3 Results

### 3.1 Selected Gut Microbiota Genera Associated With Phe Levels

**FIGURE 2:**
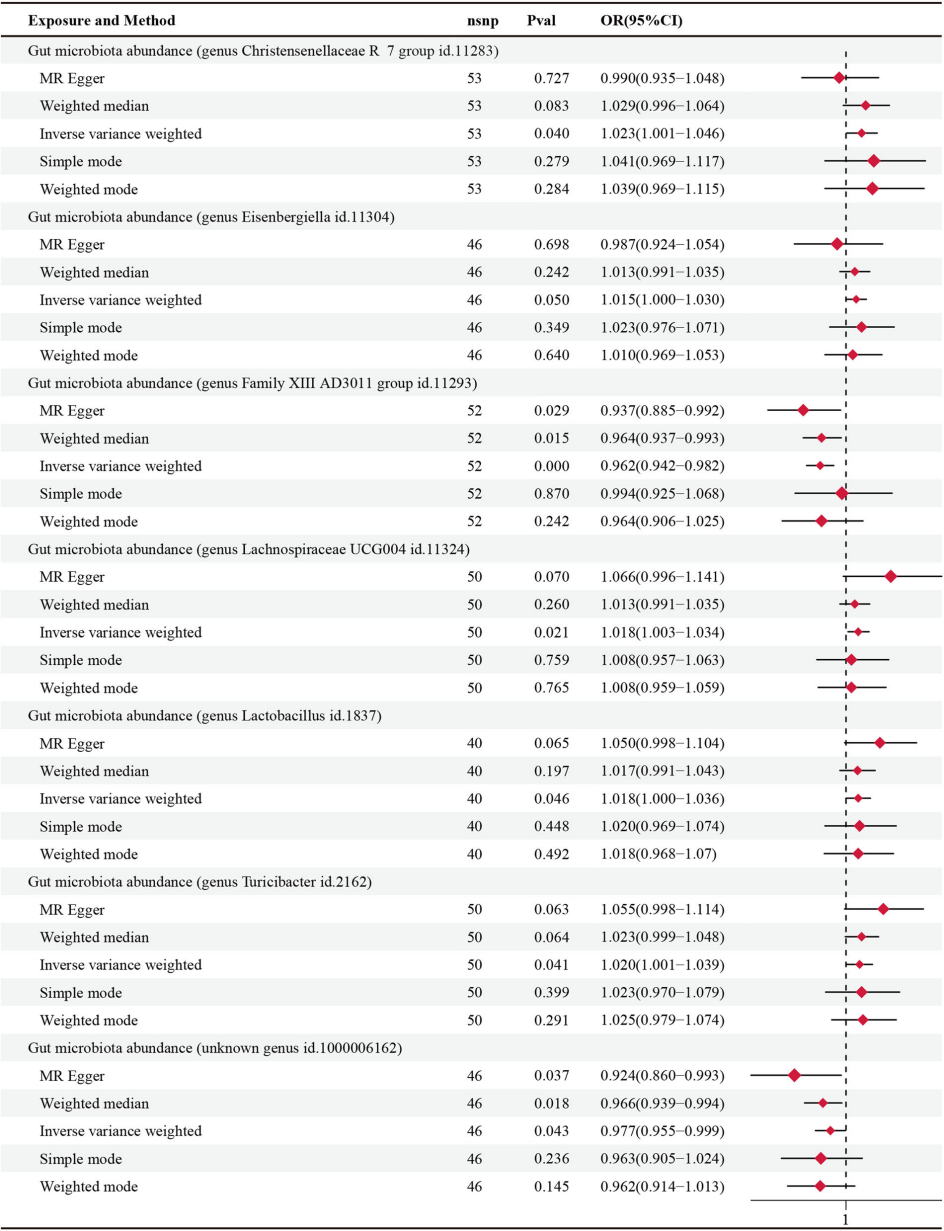
Forest plot of Mendelian randomization estimation of 7 gut microbiota and phenylalanine levels. CI, confidence interval; ID, identifier; OR, odds ratio; nSNP, the number of SNPs.

**FIGURE 3:**
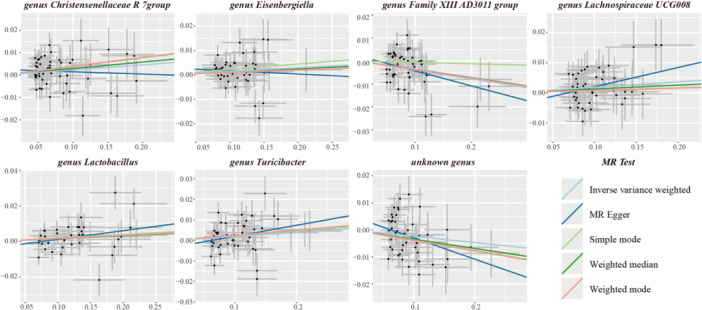
Scatter plots for the causal association between 7 gut microbiota and phenylalanine levels. Each panel represents one microbiota trait. The *X*‐axis shows the effect size of each genetic instrumental variable on the exposure. The *Y*‐axis shows the effect size of the same single‐nucleotide polymorphism (SNP) on the outcome. Each data point represents an individual SNP. The slope of the fitted line represents the causal effect estimate derived from the inverse‐variance weighted (IVW) method.

Table S2 summarized the potential association between exposure and Phe levels. MR analysis identified 7 gut microbiota genera that were significantly associated with Phe levels, which included *Family XIII AD3011 group* (a less well‐characterized classification within the Firmicutes phylum) (id: 11293), *Christensenellaceae R‐7 group* (id: 11283), *Eisenbergiella* (id: 11304), *Lachnospiraceae UCG004* (id: 11324), *Lactobacillus* (id: 1837), *Turicibacter* (id: 2162) and other unknown genera (id: 1000006162) (Table 1, Figures 2 and 3).

**TABLE 1:** IVW Results of Mendelian analysis of the association between 7 gut microbiota and phenylalanine levels.

Exposure ID	Exposure	nSNP	se	*p‐*value	OR	Adjusted *p‐*value	*F*‐statistics
ebi‐a‐GCST90016978	Gut microbiota abundance (genus *Christensenellaceae R 7 group* id.11283)	53	0.011	0.040	1.023	0.842	18.290
ebi‐a‐GCST90016991	Gut microbiota abundance (genus *Eisenbergiella* id.11304)	46	0.007	0.050	1.015	0.842	18.400
ebi‐a‐GCST90017008	Gut microbiota abundance (genus *Family XIII AD3011 group* id.11293)	52	0.010	0.000	0.962	0.027	18.614
ebi‐a‐GCST90017027	Gut microbiota abundance (genus *Lachnospiraceae UCG004* id.11324)	50	0.008	0.021	1.018	0.842	18.510
ebi‐a‐GCST90017030	Gut microbiota abundance (genus *Lactobacillus* id.1837)	40	0.009	0.046	1.018	0.842	18.544
ebi‐a‐GCST90017074	Gut microbiota abundance (genus *Turicibacter* id.2162)	50	0.009	0.041	1.020	0.842	18.923
ebi‐a‐GCST90017081	Gut microbiota abundance (unknown genus id.1000006162)	46	0.012	0.043	0.977	0.842	18.977

Among the seven selected groups, *Family XIII AD3011 group* exerted a protective influence on Phe levels (OR = 0.962, 95% CI: 0.942–0.982, *p* < 0.001), and these associations remained significant after FDR correction (adjusted *p* = 0.027) (Figure 2, Table 1). It seemed that the unknown genus also showed a protective influence on Phe levels (OR = 0.977, 95% CI: 0.955–0.999, *p* = 0.043), but the significance of the association's effects diminished upon FDR correction (adjusted *p* > 0.05). The other five groups, including *Christensenellaceae R7 group* (OR = 1.023, 95% CI: 1.001–1.046, *p* = 0.04), *Eisenbergiella* (OR = 1.015, 95% CI: 1.000–1.030, *p* = 0.050), *Lachnospiraceae UCG004* (OR = 1.018, 95% CI: 1.003–1.034, *p* = 0.021), *Lactobacillus* (OR = 1.018, 95% CI: 1.000–1.036, *p* = 0.046), *Turicibacterium* (OR = 1.020, 95% CI: 1.001–1.039, *p* = 0.041) suggested an increased risk of Phe levels, but these results did not remain significanct following FDR correction (Table 1).

### 3.2 Heterogeneity Analysis

**FIGURE 4:**
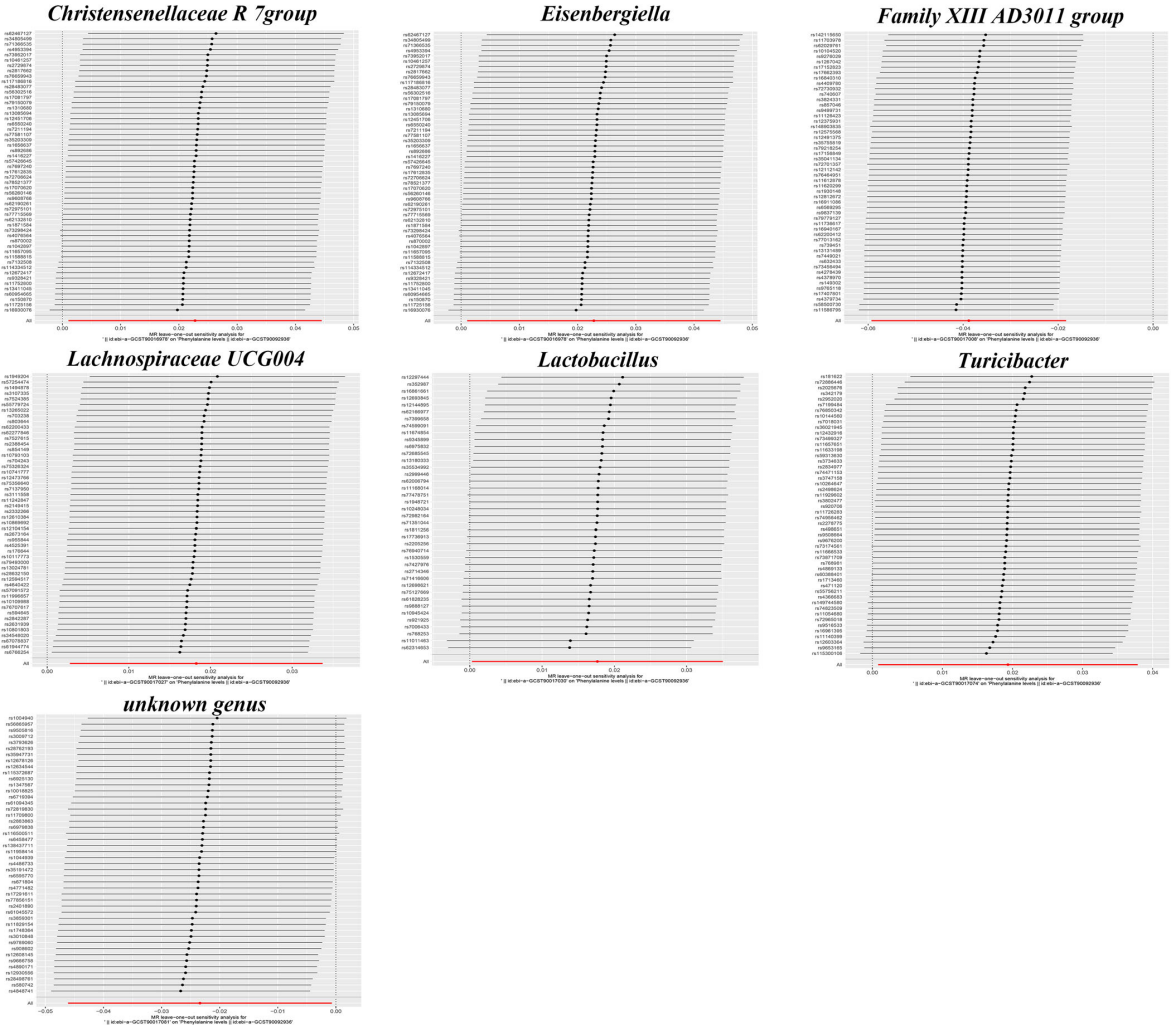
Leave‐one‐out plots for the causal association between *Family XIII AD3011 group* and phenylalanine levels. To identify influential outliers, each single‐nucleotide polymorphism (SNP) is iteratively removed from the analysis. The stability of the association is then assessed by examining the effect estimates (Beta) and confidence intervals (CIs) of the remaining SNPs. A robust result is indicated when the recalculated Beta and its CI remain on the same side of the null value (*β* = 0). If the CI crosses null after removal of a SNP, the variant is considered an influential outlier, suggesting the overall findings may not be robust.

**FIGURE 5:**
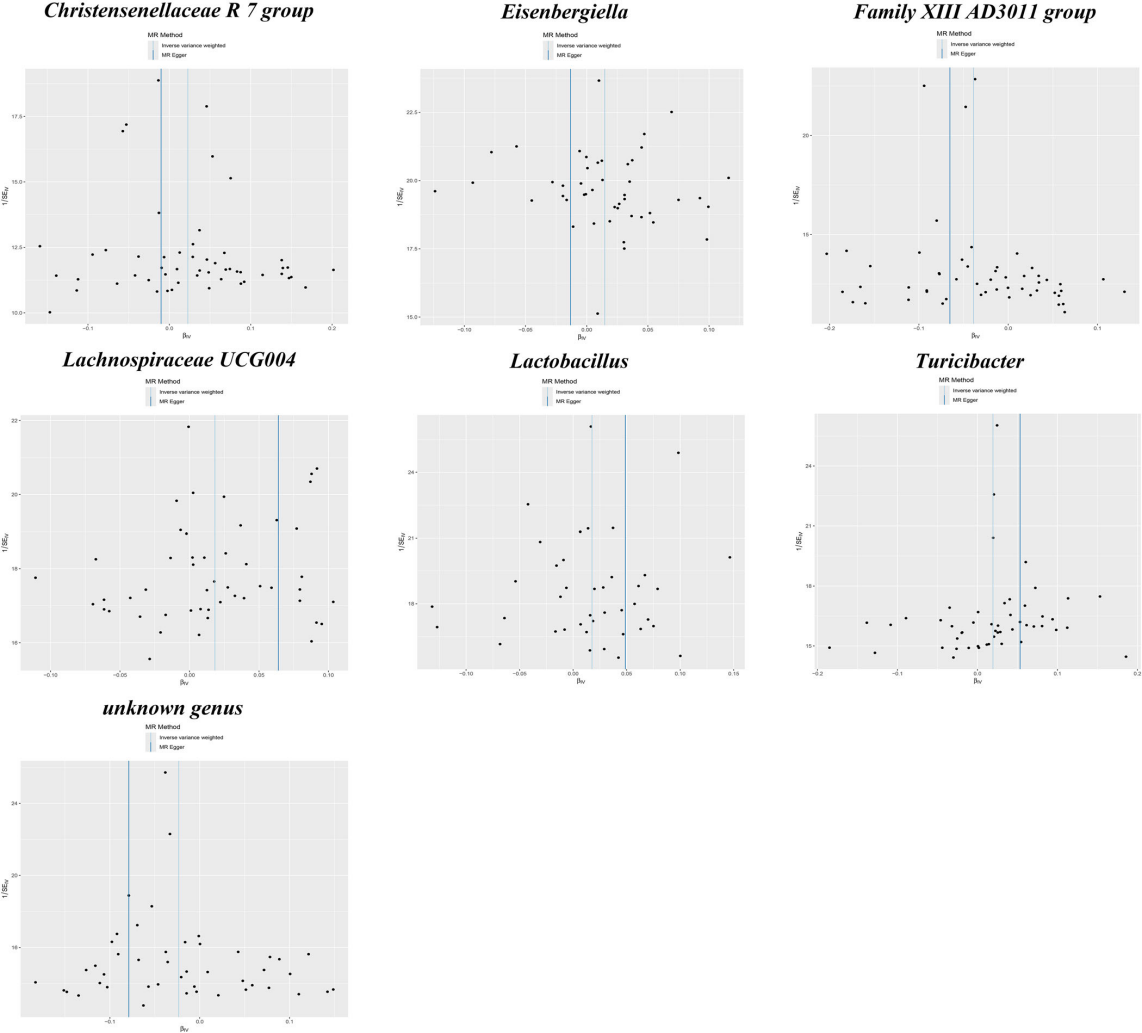
Funnel plots for the causal association between *Family XIII AD3011 group* and phenylalanine levels. This plot assesses potential bias in the causal estimate. Each point represents the causal estimate of an SNP. A symmetrical distribution suggests a lower likelihood of bias, while asymmetry indicates potential directional pleiotropy. SNP, single‐nucleotide polymorphism.

For the seven identifiedcausal relationships, the F statistics of IVs range from 17.820 to 19.523, indicating sufficient strength and minimizing potential bias from weak IVs. The results of Cochran's IVW Q test for all exposure factors showed that these IVs exhibited no notable significant heterogeneity (Table S3). Figures 4 and 5 illustrate the results of the leave‐one‐out observation and heterogeneity results for the Family *XIII AD3011* group. This was consistent with the results of Cochran's IVW Q test.

### 3.3 Horizontal Pleiotropy Analysis

No evidence of horizontal pleiotropy was observed based on the MR‐Egger intercept analysis (Table S4), indicating that the IVs did not significantly affect the results through pathways other than exposure.

## 4 Discussion

To our knowledge, this is the first two‐sample MR analysis to investigate a potential causal link between gut microbiota and Phe levels using large‐scale GWAS summary statistics. Our findings revealed several specific microbial genera associated with Phe levels, enhancing our understanding of the role of gut microbiota in PKU and suggesting possible directions for novel therapeutic strategies.

Our data showed that seven gut microbiota genera were ultimately identified as being associated with Phe levels. The *Family XIII AD3011 group* and the other unknown genera exhibited a protective effect on Phe levels, while the other five genera indicated a high risk of elevated Phe levels. However, only the *Family XIII AD3011* group maintained a significant protective effect after FDR correction. It is worth noting that, with the exception of the unknown genus, the remaining six genera were from the phylum *Firmicutes*. The human intestinal microbiota is primarily composed of four major phyla (i.e., *Firmicutes, Bacteroidetes, Actinobacteria*, and *Proteobacteria*) with the *Firmicutes* and *Bacteroidetes* accounting for 90% of the total microbial population (Sasso et al. 2023). Although the composition of gut microbiota varies among individuals, the *Firmicutes* (such as *Clostridium, Enterococcus, Lactobacillus*, and *Ruminococcus* genera) can make up to 60% of the gut microbiota. Previous research has revealed notable alterations in the gut microbiota of individuals with PKU relative to healthy controls, especially within the *Firmicutes* group. In particular, children with PKU have shown a marked reduction in *Faecalibacterium* spp., which belong to the *Ruminococcaceae* family under *Firmicutes* (Bassanini et al. 2019).

Previous observational studies have identified notable alterations in the gut microbiota of PKU patients undergoing dietary treatment. Bassanini et al. (2019) found that the most relevant alterations in the gut microbiota of PKU patients were observed within the *Firmicutes* phylum. Pinheiro de Oliveira et al. (2016) found that *Bacteroidetes* and *Firmicutes* were the most common phylum discovered, while PKU patients showed an increase in *Prevotella, Akkermansia*, and *Peptostreptococcicee*. Su et al. (2021) identified a markedly low abundance of *Bacteroides* in individuals with PKU, revealing an inverse relationship between *Bacteroides* and blood Phe concentrations. These results pointed to a potential relationship between gut microbiota and Phe levels. Many advances have been made in studying the gut microbiota, mainly due to its complex interactions with environmental factors, as well as its susceptibility to influences such as dietary patterns, drug intake, aging, and host genetics. In this sense, this highlights the need to explore gut microbiota composition in patients with PKU, as this composition may have a positive impact on the nutrient metabolism. Additionally, research has indicated the involvement of gut microbiota in Phe metabolism. For instance, Sim et al. (2015) demonstrated that Phe is converted into phenethylamine mediated by decarboxylase in gut microbiota such as *Morganella morganii*.

**FIGURE 6:**
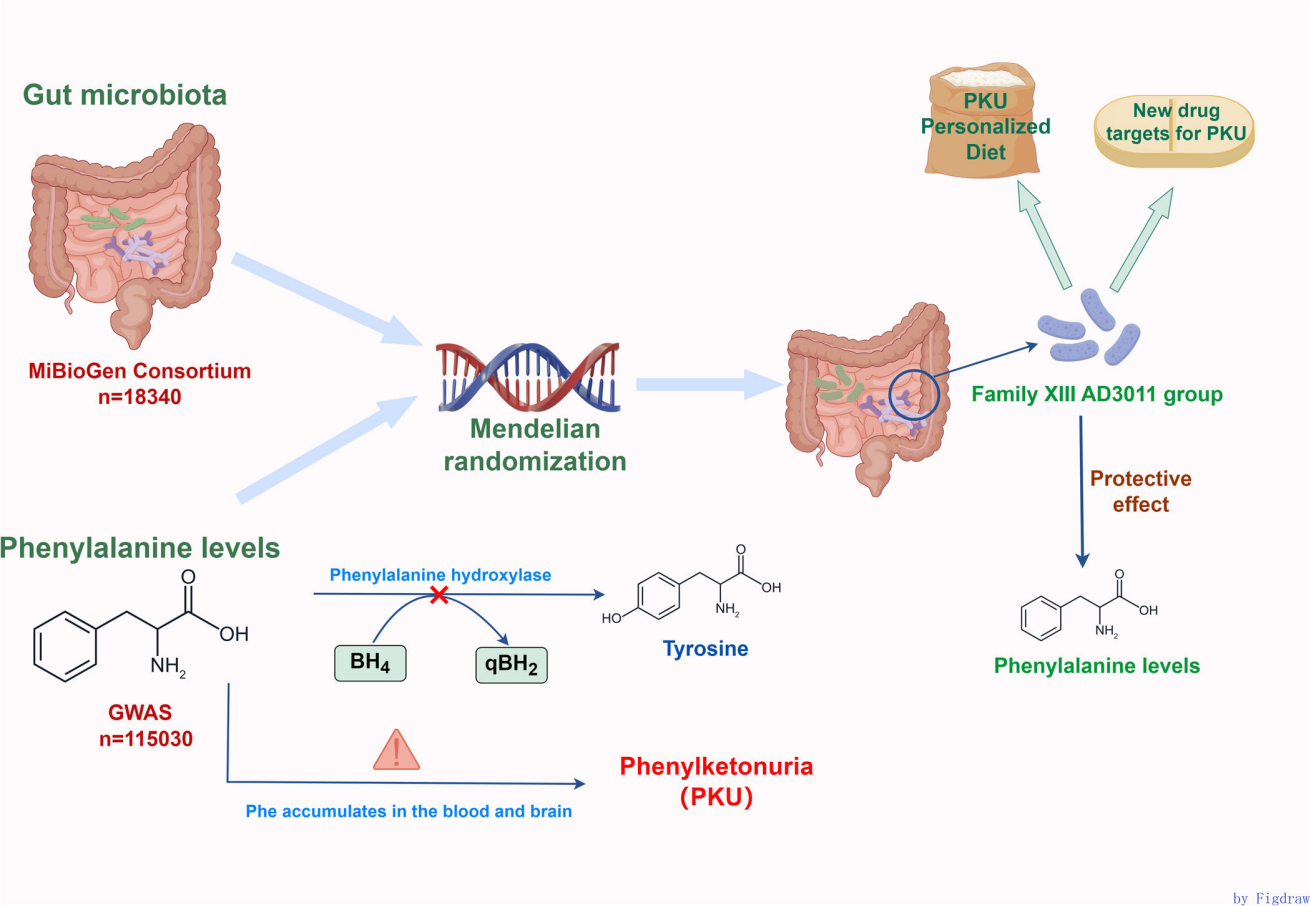
Association diagram of *Family XIII AD3011 group* and decreased phenylalanine levels by Mendelian randomization analysis.

It has been shown that *Family XIII AD3011 group* is associated with many diseases. Although this genus has been previously linked to conditions such as aortic aneurysm (Lv et al. 2024), chronic hepatitis B (Zhang et al. 2023), and respiratory infections (Huang et al. 2023), our study is the first to report its protective association with Phe levels (Figure 6). The *Family XIII AD3011 group* belongs to the *Anaerovoracaceae* family within the Clostridia class of the *Firmicutes* phylum (Guo et al. 2022). It is able to produce short‐chain fatty acid (SCFA), such as butyrate (Guo and Zhang 2024). One of the most relevant therapeutic approaches for microbiome regulation is to restore the levels of SCFA (Fusco et al. 2023). They are involved in modulating energy metabolism and supporting intestinal barrier function (Canani 2011; Guilloteau et al. 2010). Two studies had shown that butyrate can also regulate gene expression by inhibiting histone deacetylase activity (Mohamed Elfadil et al. 2023; Steliou et al. 2012), possibly including the gene for PAH. Therefore, the *Family XIII AD3011 group* may indirectly reduce Phe levels in the blood by increasing butyrate production.

Currently, the primary therapeutic approach for PKU involves maintaining a strict low‐Phe diet, which not only imposes great life pressure on patients, but also may lead to malnutrition (Al Hafid and Christodoulou 2015). Despite this, significant progress has been made in the study of protein alternatives. For instance, glycomacropeptide (GMP), derived from whey protein, is Phe‐free. It contains minimal amounts of neutral amino acids such as tryptophan, leucine, and methionine. Therefore, such treatment may lead to overloading of certain nutrients and nutritional deficiencies (van Spronsen et al. 2021). In addition, PKU can also be treated with sapropterin dihydrochloride and pegvaliase. Sapropterin dihydrochloride, a cofactor that enhances PAH enzyme activity, has been shown to lower blood phenylalanine concentrations in individuals with BH_4_‐responsive PKU. Clinical data suggest that roughly 30%–50% of these patients benefit from this pharmacological agent alongside dietary management (Longo et al. 2015a, 2015b). Recently, pegvaliase has emerged as a therapeutic option for adult patients, which utilizes a pegylated form of phenylalanine ammonia‐lyase to enzymatically degrade phenylalanine through systemic administration. However, it can trigger severe immune reactions and anaphylactic shock (Bell et al. 2017). Reducing Phe levels through modulation of the gut microbiota may offer PKU patients a more natural and effective lifestyle intervention strategy.

Nonetheless, some limitations should be noted. Due to the nature of MR design, the data derived from public resources are not individual‐level statistics, which may lead to some bias in causal estimation. Moreover, the validity of MR analysis depends on its three core assumptions, which are inherently challenging to fully meet, even with stringent quality filtering and sensitivity analyses. In addition, due to the limited number of SNPs that reached the conventional genome‐wide significance threshold (*p* < 5 × 10^−8^), we adopted a less stringent threshold (*p* < 1.5 × 10^−5^) for inclusion across the entire locus. Furthermore, although MR provides valuable insights into potential causal directions between exposure and outcome traits, it may not offer precise effect size estimations, necessitating further research for validation. Finally, because the analysis was conducted using data from individuals of European ancestry, the applicability and generaliztion of our findings to other populations may be limited.

## 5 Conclusions

Based on a two‐sample MR analysis using publicly available GWAS summary statistics, we evaluated the potential association between gut microbiota and Phe levels. Our data showed that *Family XIII AD3011 group* was associated with reduced Phe levels. This provides valuable evidence for elucidating the interaction between gut microbiota and Phe levels, offering a scientific basis for developing personalized diet and nutrition recommendations for PKU patients. Additionally, this study may also help in screening gut microbial‐based metabolites and markers as new drug targets for PKU treatment.

## Author Contributions

**Zhijuan Liu:** methodology, formal analysis, writing – original draft. **Minggang Ye:** investigation, writing – review and editing. **Huiya Jin:** investigation, writing – review and editing. **Weixia Chen:** investigation, writing – review and editing. **Hasitiyaer Jieens:** investigation, writing – review and editing. **Rui Han:** conceptualization, project administration, writing – review and editing.

## Ethics Statement

This study is a Mendelian randomization analysis based on publicly available databases, where all patients' information is fully deidentified. Because the information in the databases does not require the patient's explicit consent, the study is waived from ethical approval. The informed patient consent is not required due to the retrospective nature of the study.

## Conflicts of Interest

The authors declare no conflicts of interest.

## Supporting information

Supplementary **Table 1:**

**Table S1:** IVW Results of Mendelian analysis of the association between gut microbiota and phenylalanine levels.

**Table S2:** The heterogeneity of gut microbiota instrumental variables.

**Table S3:** The pleiotropy of gut microbiota instrumental variables.

## Data Availability

All data generated or analyzed during this study are included in this published article and its supplementary information files.
