# Supplementary material for: Relationship Between Gut Microbiota and Phenylalanine Levels: A Mendelian Randomization Study
Source: Microbiologyopen. 2025 Nov 7;14(6):e70148. doi: 10.1002/mbo3.70148 (PMC12592860; doi:10.1002/mbo3.70148)
Supplement: Supplementary file 2 — Table S1: IVW Results of Mendelian analysis of the association between gut microbiota and phenylalanine levels. [file MBO3-14-e70148-s001.docx]

**Table S1 IVW Results of Mendelian analysis of the association between gut microbiota and phenylalanine levels.**

| id.exposure | Exposure | nsnp | se | pval | OR | p.adjust | F-statistics |
| --- | --- | --- | --- | --- | --- | --- | --- |
| ebi-a-GCST90016959 | Gut microbiota abundance (genus Actinomyces id.423) | 43 | 0.009 | 0.062 | 0.984 | 0.842 | 17.951 |
| ebi-a-GCST90016960 | Gut microbiota abundance (genus Adlercreutzia id.812) | 41 | 0.010 | 0.323 | 0.990 | 0.978 | 18.631 |
| ebi-a-GCST90016961 | Gut microbiota abundance (genus Akkermansia id.4037) | 58 | 0.010 | 0.281 | 0.989 | 0.978 | 18.429 |
| ebi-a-GCST90016962 | Gut microbiota abundance (genus Alistipes id.968) | 55 | 0.012 | 0.150 | 1.017 | 0.978 | 18.284 |
| ebi-a-GCST90016963 | Gut microbiota abundance (genus Allisonella id.2174) | 39 | 0.006 | 0.961 | 1.000 | 0.987 | 18.352 |
| ebi-a-GCST90016964 | Gut microbiota abundance (genus Alloprevotella id.961) | 33 | 0.007 | 0.810 | 1.002 | 0.978 | 18.533 |
| ebi-a-GCST90016965 | Gut microbiota abundance (genus Anaerofilum id.2053) | 43 | 0.009 | 0.385 | 0.992 | 0.978 | 18.829 |
| ebi-a-GCST90016966 | Gut microbiota abundance (genus Anaerostipes id.1991) | 71 | 0.010 | 0.782 | 1.003 | 0.978 | 18.141 |
| ebi-a-GCST90016967 | Gut microbiota abundance (genus Anaerotruncus id.2054) | 50 | 0.012 | 0.504 | 0.992 | 0.978 | 18.400 |
| ebi-a-GCST90016968 | Gut microbiota abundance (genus Bacteroides id.918) | 51 | 0.012 | 0.903 | 0.999 | 0.978 | 18.310 |
| ebi-a-GCST90016969 | Gut microbiota abundance (genus Barnesiella id.944) | 44 | 0.012 | 0.150 | 1.018 | 0.978 | 19.007 |
| ebi-a-GCST90016970 | Gut microbiota abundance (genus Bifidobacterium id.436) | 59 | 0.010 | 0.382 | 0.991 | 0.978 | 19.523 |
| ebi-a-GCST90016971 | Gut microbiota abundance (genus Bilophila id.3170) | 53 | 0.011 | 0.831 | 1.002 | 0.978 | 18.533 |
| ebi-a-GCST90016972 | Gut microbiota abundance (genus Blautia id.1992) | 5 | 0.032 | 0.498 | 0.979 | 0.978 | 18.367 |
| ebi-a-GCST90016973 | Gut microbiota abundance (genus Butyricicoccus id.2055) | 45 | 0.012 | 0.890 | 1.002 | 0.978 | 18.101 |
| ebi-a-GCST90016974 | Gut microbiota abundance (genus Butyricimonas id.945) | 52 | 0.009 | 0.636 | 0.996 | 0.978 | 18.809 |
| ebi-a-GCST90016975 | Gut microbiota abundance (genus Butyrivibrio id.1993) | 49 | 0.006 | 0.303 | 0.994 | 0.978 | 19.047 |
| ebi-a-GCST90016976 | Gut microbiota abundance (genus Candidatus Soleaferrea id.11350) | 47 | 0.008 | 0.371 | 0.993 | 0.978 | 18.649 |
| ebi-a-GCST90016977 | Gut microbiota abundance (genus Catenibacterium id.2153) | 31 | 0.008 | 0.723 | 0.997 | 0.978 | 18.153 |
| ebi-a-GCST90016978 | Gut microbiota abundance (genus Christensenellaceae R 7group id.11283) | 53 | 0.011 | 0.040 | 1.023 | 0.842 | 18.290 |
| ebi-a-GCST90016979 | Gut microbiota abundance (genus Clostridium innocuum group id.14397) | 39 | 0.008 | 0.599 | 1.004 | 0.978 | 18.508 |
| ebi-a-GCST90016980 | Gut microbiota abundance (genus Clostridium sensustricto1 id.1873) | 51 | 0.010 | 0.348 | 0.990 | 0.978 | 18.040 |
| ebi-a-GCST90016981 | Gut microbiota abundance (genus Collinsella id.815) | 47 | 0.012 | 0.535 | 1.007 | 0.978 | 18.230 |
| ebi-a-GCST90016982 | Gut microbiota abundance (genus Coprobacter id.949) | 48 | 0.009 | 0.473 | 1.006 | 0.978 | 18.487 |
| ebi-a-GCST90016983 | Gut microbiota abundance (genus Coprococcus1 id.11301) | 54 | 0.011 | 0.736 | 1.004 | 0.978 | 18.547 |
| ebi-a-GCST90016984 | Gut microbiota abundance (genus Coprococcus2 id.11302) | 43 | 0.011 | 0.685 | 1.004 | 0.978 | 18.236 |
| ebi-a-GCST90016985 | Gut microbiota abundance (genus Coprococcus3 id.11303) | 39 | 0.013 | 0.758 | 0.996 | 0.978 | 18.652 |
| ebi-a-GCST90016986 | Gut microbiota abundance (genus Defluviitaleaceae UCG011 id.11287) | 49 | 0.009 | 0.930 | 0.999 | 0.985 | 18.409 |
| ebi-a-GCST90016987 | Gut microbiota abundance (genus Desulfovibrio id.3173) | 49 | 0.009 | 0.065 | 0.983 | 0.842 | 18.410 |
| ebi-a-GCST90016988 | Gut microbiota abundance (genus Dialister id.2183) | 54 | 0.010 | 0.642 | 1.005 | 0.978 | 18.167 |
| ebi-a-GCST90016989 | Gut microbiota abundance (genus Dorea id.1997) | 56 | 0.012 | 0.585 | 0.994 | 0.978 | 18.199 |
| ebi-a-GCST90016990 | Gut microbiota abundance (genus Eggerthella id.819) | 55 | 0.007 | 0.368 | 1.006 | 0.978 | 18.324 |
| ebi-a-GCST90016991 | Gut microbiota abundance (genus Eisenbergiella id.11304) | 46 | 0.007 | 0.050 | 1.015 | 0.842 | 18.400 |
| ebi-a-GCST90016992 | Gut microbiota abundance (genus Enterorhabdus id.820) | 40 | 0.009 | 0.402 | 0.992 | 0.978 | 18.731 |
| ebi-a-GCST90016993 | Gut microbiota abundance (genus Erysipelatoclostridium id.11381) | 45 | 0.010 | 0.877 | 0.998 | 0.978 | 18.953 |
| ebi-a-GCST90016994 | Gut microbiota abundance (genus Erysipelotrichaceae UCG003 id.11384) | 4 | 0.040 | 0.781 | 0.989 | 0.978 | 18.031 |
| ebi-a-GCST90016995 | Gut microbiota abundance (genus Escherichia Shigella id.3504) | 47 | 0.011 | 0.374 | 1.010 | 0.978 | 18.447 |
| ebi-a-GCST90016996 | Gut microbiota abundance (genus Eubacterium brachy group id.11296) | 38 | 0.007 | 0.082 | 1.012 | 0.888 | 18.789 |
| ebi-a-GCST90016997 | Gut microbiota abundance (genus Eubacterium coprostanoligenes group id.11375) | 50 | 0.012 | 0.695 | 1.005 | 0.978 | 18.542 |
| ebi-a-GCST90016998 | Gut microbiota abundance (genus Eubacterium eligens group id.14372) | 46 | 0.012 | 0.528 | 1.008 | 0.978 | 18.377 |
| ebi-a-GCST90016999 | Gut microbiota abundance (genus Eubacterium fissicatena group id.14373) | 36 | 0.007 | 0.199 | 0.991 | 0.978 | 18.530 |
| ebi-a-GCST90017000 | Gut microbiota abundance (genus Eubacterium hallii group id.11338) | 49 | 0.011 | 0.749 | 1.004 | 0.978 | 18.563 |
| ebi-a-GCST90017001 | Gut microbiota abundance (genus Eubacterium nodatum group id.11297) | 48 | 0.006 | 0.272 | 0.993 | 0.978 | 18.622 |
| ebi-a-GCST90017002 | Gut microbiota abundance (genus Eubacterium oxidoreducens group id.11339) | 35 | 0.008 | 0.967 | 1.000 | 0.987 | 18.251 |
| ebi-a-GCST90017003 | Gut microbiota abundance (genus Eubacterium rectale group id.14374) | 49 | 0.011 | 0.401 | 0.990 | 0.978 | 18.410 |
| ebi-a-GCST90017004 | Gut microbiota abundance (genus Eubacterium ruminantium group id.11340) | 46 | 0.009 | 0.855 | 0.998 | 0.978 | 19.040 |
| ebi-a-GCST90017005 | Gut microbiota abundance (genus Eubacterium ventriosum group id.11341) | 59 | 0.010 | 0.829 | 1.002 | 0.978 | 18.604 |
| ebi-a-GCST90017006 | Gut microbiota abundance (genus Eubacterium xylanophilum group id.14375) | 45 | 0.011 | 0.940 | 1.001 | 0.985 | 18.375 |
| ebi-a-GCST90017007 | Gut microbiota abundance (genus Faecalibacterium id.2057) | 45 | 0.012 | 0.252 | 1.014 | 0.978 | 18.660 |
| ebi-a-GCST90017008 | Gut microbiota abundance (genus Family XIII AD3011 group id.11293) | 52 | 0.010 | 0.000 | 0.962 | 0.027 | 18.614 |
| ebi-a-GCST90017009 | Gut microbiota abundance (genus Family XIII UCG001 id.11294) | 62 | 0.009 | 0.723 | 0.997 | 0.978 | 18.159 |
| ebi-a-GCST90017010 | Gut microbiota abundance (genus Flavonifractor id.2059) | 36 | 0.012 | 0.744 | 0.996 | 0.978 | 18.443 |
| ebi-a-GCST90017011 | Gut microbiota abundance (genus Fusicatenibacter id.11305) | 60 | 0.013 | 0.180 | 0.983 | 0.978 | 18.518 |
| ebi-a-GCST90017012 | Gut microbiota abundance (genus Gordonibacter id.821) | 45 | 0.006 | 0.206 | 0.992 | 0.978 | 18.822 |
| ebi-a-GCST90017013 | Gut microbiota abundance (genus Haemophilus id.3698) | 48 | 0.010 | 0.566 | 1.006 | 0.978 | 18.760 |
| ebi-a-GCST90017014 | Gut microbiota abundance (genus Holdemanella id.11393) | 38 | 0.009 | 0.746 | 0.997 | 0.978 | 19.317 |
| ebi-a-GCST90017015 | Gut microbiota abundance (genus Holdemania id.2157) | 43 | 0.011 | 0.474 | 1.008 | 0.978 | 19.251 |
| ebi-a-GCST90017016 | Gut microbiota abundance (genus Howardella id.2000) | 39 | 0.007 | 0.327 | 0.993 | 0.978 | 18.939 |
| ebi-a-GCST90017017 | Gut microbiota abundance (genus Hungatella id.11306) | 45 | 0.007 | 0.105 | 0.989 | 0.978 | 17.820 |
| ebi-a-GCST90017018 | Gut microbiota abundance (genus Intestinibacter id.11345) | 54 | 0.013 | 0.129 | 1.020 | 0.978 | 18.754 |
| ebi-a-GCST90017019 | Gut microbiota abundance (genus Intestinimonas id.2062) | 46 | 0.010 | 0.202 | 1.013 | 0.978 | 19.052 |
| ebi-a-GCST90017020 | Gut microbiota abundance (genus Lachnoclostridium id.11308) | 61 | 0.013 | 0.830 | 1.003 | 0.978 | 18.075 |
| ebi-a-GCST90017021 | Gut microbiota abundance (genus Lachnospira id.2004) | 61 | 0.011 | 0.791 | 0.997 | 0.978 | 17.983 |
| ebi-a-GCST90017022 | Gut microbiota abundance (genus Lachnospiraceae FCS020 group id.11314) | 47 | 0.008 | 0.736 | 0.997 | 0.978 | 18.466 |
| ebi-a-GCST90017023 | Gut microbiota abundance (genus Lachnospiraceae NC2004 group id.11316) | 37 | 0.016 | 0.732 | 0.995 | 0.978 | 18.350 |
| ebi-a-GCST90017024 | Gut microbiota abundance (genus Lachnospiraceae ND3007 group id.11317) | 55 | 0.011 | 0.279 | 1.012 | 0.978 | 18.065 |
| ebi-a-GCST90017025 | Gut microbiota abundance (genus Lachnospiraceae NK4A136 group id.11319) | 51 | 0.009 | 0.561 | 1.005 | 0.978 | 18.382 |
| ebi-a-GCST90017026 | Gut microbiota abundance (genus Lachnospiraceae UCG001 id.11321) | 56 | 0.011 | 0.497 | 0.993 | 0.978 | 18.689 |
| ebi-a-GCST90017027 | Gut microbiota abundance (genus Lachnospiraceae UCG004 id.11324) | 50 | 0.008 | 0.021 | 1.018 | 0.842 | 18.510 |
| ebi-a-GCST90017028 | Gut microbiota abundance (genus Lachnospiraceae UCG008 id.11328) | 48 | 0.011 | 0.635 | 0.995 | 0.978 | 19.076 |
| ebi-a-GCST90017029 | Gut microbiota abundance (genus Lachnospiraceae UCG010 id.11330) | 9 | 0.029 | 0.696 | 0.989 | 0.978 | 18.327 |
| ebi-a-GCST90017030 | Gut microbiota abundance (genus Lactobacillus id.1837) | 40 | 0.009 | 0.046 | 1.018 | 0.842 | 18.544 |
| ebi-a-GCST90017031 | Gut microbiota abundance (genus Lactococcus id.1851) | 48 | 0.006 | 0.394 | 0.995 | 0.978 | 18.681 |
| ebi-a-GCST90017032 | Gut microbiota abundance (genus Marvinbryantia id.2005) | 50 | 0.010 | 0.388 | 0.992 | 0.978 | 18.512 |
| ebi-a-GCST90017033 | Gut microbiota abundance (genus Methanobrevibacter id.123) | 45 | 0.006 | 0.866 | 1.001 | 0.978 | 18.208 |
| ebi-a-GCST90017034 | Gut microbiota abundance (genus Odoribacter id.952) | 37 | 0.015 | 0.981 | 1.000 | 0.987 | 18.371 |
| ebi-a-GCST90017035 | Gut microbiota abundance (genus Olsenella id.822) | 36 | 0.007 | 0.325 | 0.993 | 0.978 | 18.660 |
| ebi-a-GCST90017036 | Gut microbiota abundance (genus Oscillibacter id.2063) | 50 | 0.009 | 0.814 | 0.998 | 0.978 | 18.701 |
| ebi-a-GCST90017037 | Gut microbiota abundance (genus Oscillospira id.2064) | 44 | 0.010 | 0.520 | 0.993 | 0.978 | 18.396 |
| ebi-a-GCST90017038 | Gut microbiota abundance (genus Oxalobacter id.2978) | 48 | 0.007 | 0.810 | 0.998 | 0.978 | 19.078 |
| ebi-a-GCST90017039 | Gut microbiota abundance (genus Parabacteroides id.954) | 51 | 0.012 | 0.357 | 0.989 | 0.978 | 18.244 |
| ebi-a-GCST90017040 | Gut microbiota abundance (genus Paraprevotella id.962) | 45 | 0.008 | 0.901 | 0.999 | 0.978 | 19.092 |
| ebi-a-GCST90017041 | Gut microbiota abundance (genus Parasutterella id.2892) | 44 | 0.012 | 0.474 | 1.008 | 0.978 | 19.135 |
| ebi-a-GCST90017042 | Gut microbiota abundance (genus Peptococcus id.2037) | 46 | 0.008 | 0.976 | 1.000 | 0.987 | 19.308 |
| ebi-a-GCST90017043 | Gut microbiota abundance (genus Phascolarctobacterium id.2168) | 51 | 0.012 | 0.180 | 0.984 | 0.978 | 18.378 |
| ebi-a-GCST90017044 | Gut microbiota abundance (genus Prevotella7 id.11182) | 38 | 0.008 | 0.170 | 0.989 | 0.978 | 18.547 |
| ebi-a-GCST90017045 | Gut microbiota abundance (genus Prevotella9 id.11183) | 64 | 0.009 | 0.832 | 1.002 | 0.978 | 18.395 |
| ebi-a-GCST90017046 | Gut microbiota abundance (genus Rikenellaceae RC9 gut group id.11191) | 49 | 0.006 | 0.237 | 1.007 | 0.978 | 18.514 |
| ebi-a-GCST90017047 | Gut microbiota abundance (genus Romboutsia id.11347) | 51 | 0.011 | 0.939 | 0.999 | 0.985 | 18.300 |
| ebi-a-GCST90017048 | Gut microbiota abundance (genus Roseburia id.2012) | 55 | 0.016 | 0.655 | 1.007 | 0.978 | 18.659 |
| ebi-a-GCST90017049 | Gut microbiota abundance (genus Ruminiclostridium5 id.11355) | 52 | 0.012 | 0.836 | 0.997 | 0.978 | 18.517 |
| ebi-a-GCST90017050 | Gut microbiota abundance (genus Ruminiclostridium6 id.11356) | 44 | 0.011 | 0.973 | 1.000 | 0.987 | 18.966 |
| ebi-a-GCST90017051 | Gut microbiota abundance (genus Ruminiclostridium9 id.11357) | 58 | 0.011 | 0.525 | 1.007 | 0.978 | 18.512 |
| ebi-a-GCST90017052 | Gut microbiota abundance (genus Ruminococcaceae NK4A214 group id.11358) | 51 | 0.013 | 0.883 | 1.002 | 0.978 | 18.690 |
| ebi-a-GCST90017053 | Gut microbiota abundance (genus Ruminococcaceae UCG002 id.11360) | 51 | 0.011 | 0.214 | 1.014 | 0.978 | 19.202 |
| ebi-a-GCST90017054 | Gut microbiota abundance (genus Ruminococcaceae UCG003 id.11361) | 54 | 0.010 | 0.467 | 1.008 | 0.978 | 18.438 |
| ebi-a-GCST90017055 | Gut microbiota abundance (genus Ruminococcaceae UCG004 id.11362) | 41 | 0.010 | 0.676 | 0.996 | 0.978 | 18.952 |
| ebi-a-GCST90017056 | Gut microbiota abundance (genus Ruminococcaceae UCG005 id.11363) | 62 | 0.010 | 0.428 | 1.008 | 0.978 | 18.545 |
| ebi-a-GCST90017057 | Gut microbiota abundance (genus Ruminococcaceae UCG009 id.11366) | 41 | 0.009 | 0.845 | 1.002 | 0.978 | 18.907 |
| ebi-a-GCST90017058 | Gut microbiota abundance (genus Ruminococcaceae UCG010 id.11367) | 47 | 0.011 | 0.849 | 0.998 | 0.978 | 18.193 |
| ebi-a-GCST90017059 | Gut microbiota abundance (genus Ruminococcaceae UCG011 id.11368) | 40 | 0.007 | 0.628 | 0.997 | 0.978 | 18.788 |
| ebi-a-GCST90017060 | Gut microbiota abundance (genus Ruminococcaceae UCG013 id.11370) | 54 | 0.011 | 0.594 | 0.994 | 0.978 | 18.805 |
| ebi-a-GCST90017061 | Gut microbiota abundance (genus Ruminococcaceae UCG014 id.11371) | 50 | 0.011 | 0.689 | 0.996 | 0.978 | 18.544 |
| ebi-a-GCST90017062 | Gut microbiota abundance (genus Ruminococcus gauvreauii group id.11342) | 61 | 0.011 | 0.819 | 1.003 | 0.978 | 18.601 |
| ebi-a-GCST90017063 | Gut microbiota abundance (genus Ruminococcus gnavus group id.14376) | 56 | 0.011 | 0.115 | 1.017 | 0.978 | 18.366 |
| ebi-a-GCST90017064 | Gut microbiota abundance (genus Ruminococcus torques group id.14377) | 36 | 0.013 | 0.378 | 0.989 | 0.978 | 18.446 |
| ebi-a-GCST90017065 | Gut microbiota abundance (genus Ruminococcus1 id.11373) | 57 | 0.007 | 0.709 | 1.003 | 0.978 | 18.229 |
| ebi-a-GCST90017066 | Gut microbiota abundance (genus Ruminococcus2 id.11374) | 52 | 0.012 | 0.150 | 1.017 | 0.978 | 18.238 |
| ebi-a-GCST90017067 | Gut microbiota abundance (genus Sellimonas id.14369) | 49 | 0.006 | 0.687 | 0.998 | 0.978 | 18.609 |
| ebi-a-GCST90017068 | Gut microbiota abundance (genus Senegalimassilia id.11160) | 40 | 0.011 | 0.717 | 0.996 | 0.978 | 18.227 |
| ebi-a-GCST90017069 | Gut microbiota abundance (genus Slackia id.825) | 32 | 0.010 | 0.650 | 0.995 | 0.978 | 18.613 |
| ebi-a-GCST90017070 | Gut microbiota abundance (genus Streptococcus id.1853) | 55 | 0.011 | 0.808 | 0.997 | 0.978 | 18.730 |
| ebi-a-GCST90017071 | Gut microbiota abundance (genus Subdoligranulum id.2070) | 53 | 0.012 | 0.863 | 1.002 | 0.978 | 18.528 |
| ebi-a-GCST90017072 | Gut microbiota abundance (genus Sutterella id.2896) | 44 | 0.012 | 0.753 | 0.996 | 0.978 | 18.304 |
| ebi-a-GCST90017073 | Gut microbiota abundance (genus Terrisporobacter id.11348) | 38 | 0.009 | 0.239 | 1.010 | 0.978 | 18.227 |
| ebi-a-GCST90017074 | Gut microbiota abundance (genus Turicibacter id.2162) | 50 | 0.009 | 0.041 | 1.020 | 0.842 | 18.923 |
| ebi-a-GCST90017075 | Gut microbiota abundance (genus Tyzzerella3 id.11335) | 49 | 0.007 | 0.987 | 1.000 | 0.987 | 19.206 |
| ebi-a-GCST90017076 | Gut microbiota abundance (genus Veillonella id.2198) | 63 | 0.009 | 0.939 | 1.001 | 0.985 | 18.223 |
| ebi-a-GCST90017077 | Gut microbiota abundance (unknown genus id.1000000073) | 36 | 0.008 | 0.691 | 0.997 | 0.978 | 18.708 |
| ebi-a-GCST90017078 | Gut microbiota abundance (unknown genus id.1000001215) | 47 | 0.010 | 0.080 | 0.983 | 0.888 | 19.161 |
| ebi-a-GCST90017079 | Gut microbiota abundance (unknown genus id.1000005472) | 37 | 0.011 | 0.358 | 1.010 | 0.978 | 18.519 |
| ebi-a-GCST90017080 | Gut microbiota abundance (unknown genus id.1000005479) | 54 | 0.006 | 0.898 | 1.001 | 0.978 | 18.610 |
| ebi-a-GCST90017081 | Gut microbiota abundance (unknown genus id.1000006162) | 46 | 0.012 | 0.043 | 0.977 | 0.842 | 18.977 |
| ebi-a-GCST90017082 | Gut microbiota abundance (unknown genus id.1868) | 56 | 0.009 | 0.063 | 0.984 | 0.842 | 18.671 |
| ebi-a-GCST90017083 | Gut microbiota abundance (unknown genus id.2001) | 47 | 0.009 | 0.709 | 0.997 | 0.978 | 18.333 |
| ebi-a-GCST90017084 | Gut microbiota abundance (unknown genus id.2041) | 61 | 0.010 | 0.293 | 0.989 | 0.978 | 18.477 |
| ebi-a-GCST90017085 | Gut microbiota abundance (unknown genus id.2071) | 50 | 0.009 | 0.163 | 0.988 | 0.978 | 18.695 |
| ebi-a-GCST90017086 | Gut microbiota abundance (unknown genus id.2755) | 56 | 0.012 | 0.362 | 0.989 | 0.978 | 18.698 |
| ebi-a-GCST90017087 | Gut microbiota abundance (unknown genus id.826) | 42 | 0.008 | 0.549 | 0.995 | 0.978 | 18.603 |
| ebi-a-GCST90017088 | Gut microbiota abundance (unknown genus id.959) | 68 | 0.008 | 0.785 | 1.002 | 0.978 | 19.067 |
