# Supplementary material for: Relationship Between Gut Microbiota and Phenylalanine Levels: A Mendelian Randomization Study
Source: Microbiologyopen. 2025 Nov 7;14(6):e70148. doi: 10.1002/mbo3.70148 (PMC12592860; doi:10.1002/mbo3.70148)
Supplement: Supplementary file 3 — Table S2: The heterogeneity of gut microbiota instrumental variables. [file MBO3-14-e70148-s002.docx]

Table S2 The heterogeneity of gut microbiota instrumental variables.

| id.exposure | **Exposure** | **Cochran's Q** | **df** | **P-value** |
| --- | --- | --- | --- | --- |
| ebi-a-GCST90016959 | Gut microbiota abundance (genus Actinomyces id.423) | 31.215 | 42 | 0.889 |
| ebi-a-GCST90016960 | Gut microbiota abundance (genus Adlercreutzia id.812) | 41.556 | 40 | 0.403 |
| ebi-a-GCST90016961 | Gut microbiota abundance (genus Akkermansia id.4037) | 70.084 | 57 | 0.114 |
| ebi-a-GCST90016962 | Gut microbiota abundance (genus Alistipes id.968) | 52.782 | 54 | 0.521 |
| ebi-a-GCST90016963 | Gut microbiota abundance (genus Allisonella id.2174) | 33.732 | 38 | 0.667 |
| ebi-a-GCST90016964 | Gut microbiota abundance (genus Alloprevotella id.961) | 42.201 | 32 | 0.107 |
| ebi-a-GCST90016965 | Gut microbiota abundance (genus Anaerofilum id.2053) | 69.190 | 42 | 0.005 |
| ebi-a-GCST90016966 | Gut microbiota abundance (genus Anaerostipes id.1991) | 55.586 | 70 | 0.895 |
| ebi-a-GCST90016967 | Gut microbiota abundance (genus Anaerotruncus id.2054) | 49.985 | 49 | 0.434 |
| ebi-a-GCST90016968 | Gut microbiota abundance (genus Bacteroides id.918) | 42.892 | 50 | 0.752 |
| ebi-a-GCST90016969 | Gut microbiota abundance (genus Barnesiella id.944) | 49.296 | 43 | 0.236 |
| ebi-a-GCST90016970 | Gut microbiota abundance (genus Bifidobacterium id.436) | 46.902 | 58 | 0.851 |
| ebi-a-GCST90016971 | Gut microbiota abundance (genus Bilophila id.3170) | 57.067 | 52 | 0.292 |
| ebi-a-GCST90016972 | Gut microbiota abundance (genus Blautia id.1992) | 2.758 | 4 | 0.599 |
| ebi-a-GCST90016973 | Gut microbiota abundance (genus Butyricicoccus id.2055) | 35.568 | 44 | 0.814 |
| ebi-a-GCST90016974 | Gut microbiota abundance (genus Butyricimonas id.945) | 52.832 | 51 | 0.403 |
| ebi-a-GCST90016975 | Gut microbiota abundance (genus Butyrivibrio id.1993) | 59.329 | 48 | 0.127 |
| ebi-a-GCST90016976 | Gut microbiota abundance (genus Candidatus Soleaferrea id.11350) | 41.231 | 46 | 0.672 |
| ebi-a-GCST90016977 | Gut microbiota abundance (genus Catenibacterium id.2153) | 39.777 | 30 | 0.109 |
| ebi-a-GCST90016978 | Gut microbiota abundance (genus Christensenellaceae R 7group id.11283) | 51.427 | 52 | 0.496 |
| ebi-a-GCST90016979 | Gut microbiota abundance (genus Clostridium innocuum group id.14397) | 43.277 | 38 | 0.256 |
| ebi-a-GCST90016980 | Gut microbiota abundance (genus Clostridium sensustricto1 id.1873) | 44.599 | 50 | 0.689 |
| ebi-a-GCST90016981 | Gut microbiota abundance (genus Collinsella id.815) | 51.825 | 46 | 0.257 |
| ebi-a-GCST90016982 | Gut microbiota abundance (genus Coprobacter id.949) | 56.640 | 47 | 0.158 |
| ebi-a-GCST90016983 | Gut microbiota abundance (genus Coprococcus1 id.11301) | 52.909 | 53 | 0.478 |
| ebi-a-GCST90016984 | Gut microbiota abundance (genus Coprococcus2 id.11302) | 27.419 | 42 | 0.960 |
| ebi-a-GCST90016985 | Gut microbiota abundance (genus Coprococcus3 id.11303) | 22.824 | 38 | 0.976 |
| ebi-a-GCST90016986 | Gut microbiota abundance (genus Defluviitaleaceae UCG011 id.11287) | 35.608 | 48 | 0.907 |
| ebi-a-GCST90016987 | Gut microbiota abundance (genus Desulfovibrio id.3173) | 50.386 | 48 | 0.379 |
| ebi-a-GCST90016988 | Gut microbiota abundance (genus Dialister id.2183) | 47.290 | 53 | 0.695 |
| ebi-a-GCST90016989 | Gut microbiota abundance (genus Dorea id.1997) | 61.707 | 55 | 0.249 |
| ebi-a-GCST90016990 | Gut microbiota abundance (genus Eggerthella id.819) | 55.034 | 54 | 0.435 |
| ebi-a-GCST90016991 | Gut microbiota abundance (genus Eisenbergiella id.11304) | 39.996 | 45 | 0.683 |
| ebi-a-GCST90016992 | Gut microbiota abundance (genus Enterorhabdus id.820) | 33.626 | 39 | 0.713 |
| ebi-a-GCST90016993 | Gut microbiota abundance (genus Erysipelatoclostridium id.11381) | 48.932 | 44 | 0.282 |
| ebi-a-GCST90016994 | Gut microbiota abundance (genus Erysipelotrichaceae UCG003 id.11384) | 3.516 | 3 | 0.319 |
| ebi-a-GCST90016995 | Gut microbiota abundance (genus Escherichia Shigella id.3504) | 55.877 | 46 | 0.151 |
| ebi-a-GCST90016996 | Gut microbiota abundance (genus Eubacterium brachy group id.11296) | 38.805 | 37 | 0.388 |
| ebi-a-GCST90016997 | Gut microbiota abundance (genus Eubacterium coprostanoligenes group id.11375) | 46.261 | 49 | 0.585 |
| ebi-a-GCST90016998 | Gut microbiota abundance (genus Eubacterium eligens group id.14372) | 52.407 | 45 | 0.209 |
| ebi-a-GCST90016999 | Gut microbiota abundance (genus Eubacterium fissicatena group id.14373) | 32.870 | 35 | 0.571 |
| ebi-a-GCST90017000 | Gut microbiota abundance (genus Eubacterium hallii group id.11338) | 46.553 | 48 | 0.532 |
| ebi-a-GCST90017001 | Gut microbiota abundance (genus Eubacterium nodatum group id.11297) | 56.477 | 47 | 0.162 |
| ebi-a-GCST90017002 | Gut microbiota abundance (genus Eubacterium oxidoreducens group id.11339) | 20.513 | 34 | 0.967 |
| ebi-a-GCST90017003 | Gut microbiota abundance (genus Eubacterium rectale group id.14374) | 47.585 | 48 | 0.490 |
| ebi-a-GCST90017004 | Gut microbiota abundance (genus Eubacterium ruminantium group id.11340) | 46.162 | 45 | 0.424 |
| ebi-a-GCST90017005 | Gut microbiota abundance (genus Eubacterium ventriosum group id.11341) | 48.615 | 58 | 0.805 |
| ebi-a-GCST90017006 | Gut microbiota abundance (genus Eubacterium xylanophilum group id.14375) | 29.950 | 44 | 0.948 |
| ebi-a-GCST90017007 | Gut microbiota abundance (genus Faecalibacterium id.2057) | 50.471 | 44 | 0.233 |
| ebi-a-GCST90017008 | Gut microbiota abundance (genus Family XIII AD3011 group id.11293) | 52.042 | 51 | 0.433 |
| ebi-a-GCST90017009 | Gut microbiota abundance (genus Family XIII UCG001 id.11294) | 49.169 | 61 | 0.862 |
| ebi-a-GCST90017010 | Gut microbiota abundance (genus Flavonifractor id.2059) | 36.459 | 35 | 0.401 |
| ebi-a-GCST90017011 | Gut microbiota abundance (genus Fusicatenibacter id.11305) | 82.051 | 59 | 0.025 |
| ebi-a-GCST90017012 | Gut microbiota abundance (genus Gordonibacter id.821) | 40.594 | 44 | 0.618 |
| ebi-a-GCST90017013 | Gut microbiota abundance (genus Haemophilus id.3698) | 65.773 | 47 | 0.037 |
| ebi-a-GCST90017014 | Gut microbiota abundance (genus Holdemanella id.11393) | 39.867 | 37 | 0.344 |
| ebi-a-GCST90017015 | Gut microbiota abundance (genus Holdemania id.2157) | 58.420 | 42 | 0.047 |
| ebi-a-GCST90017016 | Gut microbiota abundance (genus Howardella id.2000) | 41.403 | 38 | 0.324 |
| ebi-a-GCST90017017 | Gut microbiota abundance (genus Hungatella id.11306) | 50.124 | 44 | 0.243 |
| ebi-a-GCST90017018 | Gut microbiota abundance (genus Intestinibacter id.11345) | 95.241 | 53 | 0.000 |
| ebi-a-GCST90017019 | Gut microbiota abundance (genus Intestinimonas id.2062) | 45.880 | 45 | 0.435 |
| ebi-a-GCST90017020 | Gut microbiota abundance (genus Lachnoclostridium id.11308) | 77.385 | 60 | 0.065 |
| ebi-a-GCST90017021 | Gut microbiota abundance (genus Lachnospira id.2004) | 70.493 | 60 | 0.167 |
| ebi-a-GCST90017022 | Gut microbiota abundance (genus Lachnospiraceae FCS020 group id.11314) | 41.372 | 46 | 0.666 |
| ebi-a-GCST90017023 | Gut microbiota abundance (genus Lachnospiraceae NC2004 group id.11316) | 53.687 | 36 | 0.029 |
| ebi-a-GCST90017024 | Gut microbiota abundance (genus Lachnospiraceae ND3007 group id.11317) | 56.095 | 54 | 0.396 |
| ebi-a-GCST90017025 | Gut microbiota abundance (genus Lachnospiraceae NK4A136 group id.11319) | 46.974 | 50 | 0.596 |
| ebi-a-GCST90017026 | Gut microbiota abundance (genus Lachnospiraceae UCG001 id.11321) | 59.150 | 55 | 0.327 |
| ebi-a-GCST90017027 | Gut microbiota abundance (genus Lachnospiraceae UCG004 id.11324) | 40.470 | 49 | 0.802 |
| ebi-a-GCST90017028 | Gut microbiota abundance (genus Lachnospiraceae UCG008 id.11328) | 40.091 | 47 | 0.752 |
| ebi-a-GCST90017029 | Gut microbiota abundance (genus Lachnospiraceae UCG010 id.11330) | 3.336 | 8 | 0.912 |
| ebi-a-GCST90017030 | Gut microbiota abundance (genus Lactobacillus id.1837) | 42.783 | 39 | 0.312 |
| ebi-a-GCST90017031 | Gut microbiota abundance (genus Lactococcus id.1851) | 46.338 | 47 | 0.500 |
| ebi-a-GCST90017032 | Gut microbiota abundance (genus Marvinbryantia id.2005) | 49.815 | 49 | 0.441 |
| ebi-a-GCST90017033 | Gut microbiota abundance (genus Methanobrevibacter id.123) | 33.776 | 44 | 0.868 |
| ebi-a-GCST90017034 | Gut microbiota abundance (genus Odoribacter id.952) | 44.420 | 36 | 0.158 |
| ebi-a-GCST90017035 | Gut microbiota abundance (genus Olsenella id.822) | 40.687 | 35 | 0.234 |
| ebi-a-GCST90017036 | Gut microbiota abundance (genus Oscillibacter id.2063) | 39.175 | 49 | 0.841 |
| ebi-a-GCST90017037 | Gut microbiota abundance (genus Oscillospira id.2064) | 45.754 | 43 | 0.359 |
| ebi-a-GCST90017038 | Gut microbiota abundance (genus Oxalobacter id.2978) | 51.160 | 47 | 0.314 |
| ebi-a-GCST90017039 | Gut microbiota abundance (genus Parabacteroides id.954) | 52.681 | 50 | 0.371 |
| ebi-a-GCST90017040 | Gut microbiota abundance (genus Paraprevotella id.962) | 27.571 | 44 | 0.975 |
| ebi-a-GCST90017041 | Gut microbiota abundance (genus Parasutterella id.2892) | 58.270 | 43 | 0.060 |
| ebi-a-GCST90017042 | Gut microbiota abundance (genus Peptococcus id.2037) | 58.788 | 45 | 0.081 |
| ebi-a-GCST90017043 | Gut microbiota abundance (genus Phascolarctobacterium id.2168) | 76.554 | 50 | 0.009 |
| ebi-a-GCST90017044 | Gut microbiota abundance (genus Prevotella7 id.11182) | 55.469 | 37 | 0.026 |
| ebi-a-GCST90017045 | Gut microbiota abundance (genus Prevotella9 id.11183) | 76.048 | 63 | 0.125 |
| ebi-a-GCST90017046 | Gut microbiota abundance (genus Rikenellaceae RC9 gut group id.11191) | 58.901 | 48 | 0.135 |
| ebi-a-GCST90017047 | Gut microbiota abundance (genus Romboutsia id.11347) | 58.287 | 50 | 0.197 |
| ebi-a-GCST90017048 | Gut microbiota abundance (genus Roseburia id.2012) | 115.957 | 54 | 0.000 |
| ebi-a-GCST90017049 | Gut microbiota abundance (genus Ruminiclostridium5 id.11355) | 58.720 | 51 | 0.214 |
| ebi-a-GCST90017050 | Gut microbiota abundance (genus Ruminiclostridium6 id.11356) | 41.227 | 43 | 0.548 |
| ebi-a-GCST90017051 | Gut microbiota abundance (genus Ruminiclostridium9 id.11357) | 52.107 | 57 | 0.659 |
| ebi-a-GCST90017052 | Gut microbiota abundance (genus Ruminococcaceae NK4A214 group id.11358) | 59.403 | 50 | 0.170 |
| ebi-a-GCST90017053 | Gut microbiota abundance (genus Ruminococcaceae UCG002 id.11360) | 52.605 | 50 | 0.374 |
| ebi-a-GCST90017054 | Gut microbiota abundance (genus Ruminococcaceae UCG003 id.11361) | 57.597 | 53 | 0.309 |
| ebi-a-GCST90017055 | Gut microbiota abundance (genus Ruminococcaceae UCG004 id.11362) | 42.459 | 40 | 0.366 |
| ebi-a-GCST90017056 | Gut microbiota abundance (genus Ruminococcaceae UCG005 id.11363) | 56.621 | 61 | 0.635 |
| ebi-a-GCST90017057 | Gut microbiota abundance (genus Ruminococcaceae UCG009 id.11366) | 28.051 | 40 | 0.922 |
| ebi-a-GCST90017058 | Gut microbiota abundance (genus Ruminococcaceae UCG010 id.11367) | 42.403 | 46 | 0.624 |
| ebi-a-GCST90017059 | Gut microbiota abundance (genus Ruminococcaceae UCG011 id.11368) | 48.753 | 39 | 0.136 |
| ebi-a-GCST90017060 | Gut microbiota abundance (genus Ruminococcaceae UCG013 id.11370) | 47.240 | 53 | 0.697 |
| ebi-a-GCST90017061 | Gut microbiota abundance (genus Ruminococcaceae UCG014 id.11371) | 54.171 | 49 | 0.284 |
| ebi-a-GCST90017062 | Gut microbiota abundance (genus Ruminococcus gauvreauii group id.11342) | 69.437 | 60 | 0.189 |
| ebi-a-GCST90017063 | Gut microbiota abundance (genus Ruminococcus gnavus group id.14376) | 50.661 | 55 | 0.641 |
| ebi-a-GCST90017064 | Gut microbiota abundance (genus Ruminococcus torques group id.14377) | 31.813 | 35 | 0.623 |
| ebi-a-GCST90017065 | Gut microbiota abundance (genus Ruminococcus1 id.11373) | 49.834 | 56 | 0.706 |
| ebi-a-GCST90017066 | Gut microbiota abundance (genus Ruminococcus2 id.11374) | 40.254 | 51 | 0.860 |
| ebi-a-GCST90017067 | Gut microbiota abundance (genus Sellimonas id.14369) | 60.087 | 48 | 0.113 |
| ebi-a-GCST90017068 | Gut microbiota abundance (genus Senegalimassilia id.11160) | 53.617 | 39 | 0.060 |
| ebi-a-GCST90017069 | Gut microbiota abundance (genus Slackia id.825) | 37.717 | 31 | 0.189 |
| ebi-a-GCST90017070 | Gut microbiota abundance (genus Streptococcus id.1853) | 53.646 | 54 | 0.488 |
| ebi-a-GCST90017071 | Gut microbiota abundance (genus Subdoligranulum id.2070) | 34.671 | 52 | 0.969 |
| ebi-a-GCST90017072 | Gut microbiota abundance (genus Sutterella id.2896) | 46.119 | 43 | 0.345 |
| ebi-a-GCST90017073 | Gut microbiota abundance (genus Terrisporobacter id.11348) | 31.382 | 37 | 0.729 |
| ebi-a-GCST90017074 | Gut microbiota abundance (genus Turicibacter id.2162) | 59.609 | 49 | 0.143 |
| ebi-a-GCST90017075 | Gut microbiota abundance (genus Tyzzerella3 id.11335) | 41.181 | 48 | 0.746 |
| ebi-a-GCST90017076 | Gut microbiota abundance (genus Veillonella id.2198) | 59.544 | 62 | 0.565 |
| ebi-a-GCST90017077 | Gut microbiota abundance (unknown genus id.1000000073) | 35.720 | 35 | 0.434 |
| ebi-a-GCST90017078 | Gut microbiota abundance (unknown genus id.1000001215) | 32.981 | 46 | 0.925 |
| ebi-a-GCST90017079 | Gut microbiota abundance (unknown genus id.1000005472) | 46.857 | 36 | 0.106 |
| ebi-a-GCST90017080 | Gut microbiota abundance (unknown genus id.1000005479) | 44.103 | 53 | 0.803 |
| ebi-a-GCST90017081 | Gut microbiota abundance (unknown genus id.1000006162) | 65.680 | 45 | 0.024 |
| ebi-a-GCST90017082 | Gut microbiota abundance (unknown genus id.1868) | 53.239 | 55 | 0.542 |
| ebi-a-GCST90017083 | Gut microbiota abundance (unknown genus id.2001) | 35.560 | 46 | 0.867 |
| ebi-a-GCST90017084 | Gut microbiota abundance (unknown genus id.2041) | 69.191 | 60 | 0.195 |
| ebi-a-GCST90017085 | Gut microbiota abundance (unknown genus id.2071) | 41.289 | 49 | 0.775 |
| ebi-a-GCST90017086 | Gut microbiota abundance (unknown genus id.2755) | 77.649 | 55 | 0.024 |
| ebi-a-GCST90017087 | Gut microbiota abundance (unknown genus id.826) | 56.764 | 41 | 0.052 |
| ebi-a-GCST90017088 | Gut microbiota abundance (unknown genus id.959) | 63.570 | 67 | 0.596 |
