# Supplementary material for: Relationship Between Gut Microbiota and Phenylalanine Levels: A Mendelian Randomization Study
Source: Microbiologyopen. 2025 Nov 7;14(6):e70148. doi: 10.1002/mbo3.70148 (PMC12592860; doi:10.1002/mbo3.70148)
Supplement: Supplementary file 4 — Table S3: The pleiotropy of gut microbiota instrumental variables. [file MBO3-14-e70148-s004.docx]

Table S3 The pleiotropy of gut microbiota instrumental variables.

| id.exposure | **Exposure** | **Egger_intercept** | **Pval** |
| --- | --- | --- | --- |
| ebi-a-GCST90016959 | Gut microbiota abundance (genus Actinomyces id.423) | -0.001 | 0.799 |
| ebi-a-GCST90016960 | Gut microbiota abundance (genus Adlercreutzia id.812) | 0.001 | 0.736 |
| ebi-a-GCST90016961 | Gut microbiota abundance (genus Akkermansia id.4037) | 0.003 | 0.184 |
| ebi-a-GCST90016962 | Gut microbiota abundance (genus Alistipes id.968) | 0.002 | 0.436 |
| ebi-a-GCST90016963 | Gut microbiota abundance (genus Allisonella id.2174) | 0.002 | 0.709 |
| ebi-a-GCST90016964 | Gut microbiota abundance (genus Alloprevotella id.961) | 0.010 | 0.118 |
| ebi-a-GCST90016965 | Gut microbiota abundance (genus Anaerofilum id.2053) | -0.004 | 0.394 |
| ebi-a-GCST90016966 | Gut microbiota abundance (genus Anaerostipes id.1991) | -0.002 | 0.244 |
| ebi-a-GCST90016967 | Gut microbiota abundance (genus Anaerotruncus id.2054) | 0.001 | 0.564 |
| ebi-a-GCST90016968 | Gut microbiota abundance (genus Bacteroides id.918) | 0.000 | 0.863 |
| ebi-a-GCST90016969 | Gut microbiota abundance (genus Barnesiella id.944) | 0.000 | 0.883 |
| ebi-a-GCST90016970 | Gut microbiota abundance (genus Bifidobacterium id.436) | 0.003 | 0.122 |
| ebi-a-GCST90016971 | Gut microbiota abundance (genus Bilophila id.3170) | 0.002 | 0.410 |
| ebi-a-GCST90016972 | Gut microbiota abundance (genus Blautia id.1992) | 0.003 | 0.544 |
| ebi-a-GCST90016973 | Gut microbiota abundance (genus Butyricicoccus id.2055) | 0.001 | 0.759 |
| ebi-a-GCST90016974 | Gut microbiota abundance (genus Butyricimonas id.945) | -0.002 | 0.518 |
| ebi-a-GCST90016975 | Gut microbiota abundance (genus Butyrivibrio id.1993) | -0.005 | 0.128 |
| ebi-a-GCST90016976 | Gut microbiota abundance (genus Candidatus Soleaferrea id.11350) | -0.001 | 0.726 |
| ebi-a-GCST90016977 | Gut microbiota abundance (genus Catenibacterium id.2153) | -0.001 | 0.859 |
| ebi-a-GCST90016978 | Gut microbiota abundance (genus Christensenellaceae R 7group id.11283) | 0.002 | 0.225 |
| ebi-a-GCST90016979 | Gut microbiota abundance (genus Clostridium innocuum group id.14397) | -0.003 | 0.458 |
| ebi-a-GCST90016980 | Gut microbiota abundance (genus Clostridium sensustricto1 id.1873) | 0.001 | 0.587 |
| ebi-a-GCST90016981 | Gut microbiota abundance (genus Collinsella id.815) | 0.001 | 0.776 |
| ebi-a-GCST90016982 | Gut microbiota abundance (genus Coprobacter id.949) | 0.000 | 0.894 |
| ebi-a-GCST90016983 | Gut microbiota abundance (genus Coprococcus1 id.11301) | 0.003 | 0.142 |
| ebi-a-GCST90016984 | Gut microbiota abundance (genus Coprococcus2 id.11302) | 0.004 | 0.169 |
| ebi-a-GCST90016985 | Gut microbiota abundance (genus Coprococcus3 id.11303) | 0.001 | 0.689 |
| ebi-a-GCST90016986 | Gut microbiota abundance (genus Defluviitaleaceae UCG011 id.11287) | 0.000 | 0.974 |
| ebi-a-GCST90016987 | Gut microbiota abundance (genus Desulfovibrio id.3173) | 0.002 | 0.525 |
| ebi-a-GCST90016988 | Gut microbiota abundance (genus Dialister id.2183) | -0.001 | 0.647 |
| ebi-a-GCST90016989 | Gut microbiota abundance (genus Dorea id.1997) | 0.000 | 0.871 |
| ebi-a-GCST90016990 | Gut microbiota abundance (genus Eggerthella id.819) | 0.000 | 0.881 |
| ebi-a-GCST90016991 | Gut microbiota abundance (genus Eisenbergiella id.11304) | 0.003 | 0.401 |
| ebi-a-GCST90016992 | Gut microbiota abundance (genus Enterorhabdus id.820) | -0.002 | 0.403 |
| ebi-a-GCST90016993 | Gut microbiota abundance (genus Erysipelatoclostridium id.11381) | 0.005 | 0.099 |
| ebi-a-GCST90016994 | Gut microbiota abundance (genus Erysipelotrichaceae UCG003 id.11384) | -0.003 | 0.797 |
| ebi-a-GCST90016995 | Gut microbiota abundance (genus Escherichia Shigella id.3504) | -0.001 | 0.581 |
| ebi-a-GCST90016996 | Gut microbiota abundance (genus Eubacterium brachy group id.11296) | 0.001 | 0.865 |
| ebi-a-GCST90016997 | Gut microbiota abundance (genus Eubacterium coprostanoligenes group id.11375) | 0.002 | 0.464 |
| ebi-a-GCST90016998 | Gut microbiota abundance (genus Eubacterium eligens group id.14372) | 0.001 | 0.564 |
| ebi-a-GCST90016999 | Gut microbiota abundance (genus Eubacterium fissicatena group id.14373) | 0.001 | 0.878 |
| ebi-a-GCST90017000 | Gut microbiota abundance (genus Eubacterium hallii group id.11338) | 0.001 | 0.648 |
| ebi-a-GCST90017001 | Gut microbiota abundance (genus Eubacterium nodatum group id.11297) | -0.002 | 0.588 |
| ebi-a-GCST90017002 | Gut microbiota abundance (genus Eubacterium oxidoreducens group id.11339) | 0.000 | 0.965 |
| ebi-a-GCST90017003 | Gut microbiota abundance (genus Eubacterium rectale group id.14374) | 0.001 | 0.529 |
| ebi-a-GCST90017004 | Gut microbiota abundance (genus Eubacterium ruminantium group id.11340) | 0.003 | 0.413 |
| ebi-a-GCST90017005 | Gut microbiota abundance (genus Eubacterium ventriosum group id.11341) | 0.003 | 0.156 |
| ebi-a-GCST90017006 | Gut microbiota abundance (genus Eubacterium xylanophilum group id.14375) | -0.001 | 0.601 |
| ebi-a-GCST90017007 | Gut microbiota abundance (genus Faecalibacterium id.2057) | 0.001 | 0.470 |
| ebi-a-GCST90017008 | Gut microbiota abundance (genus Family XIII AD3011 group id.11293) | 0.002 | 0.335 |
| ebi-a-GCST90017009 | Gut microbiota abundance (genus Family XIII UCG001 id.11294) | 0.002 | 0.416 |
| ebi-a-GCST90017010 | Gut microbiota abundance (genus Flavonifractor id.2059) | 0.003 | 0.403 |
| ebi-a-GCST90017011 | Gut microbiota abundance (genus Fusicatenibacter id.11305) | -0.003 | 0.210 |
| ebi-a-GCST90017012 | Gut microbiota abundance (genus Gordonibacter id.821) | -0.002 | 0.678 |
| ebi-a-GCST90017013 | Gut microbiota abundance (genus Haemophilus id.3698) | 0.000 | 0.972 |
| ebi-a-GCST90017014 | Gut microbiota abundance (genus Holdemanella id.11393) | -0.001 | 0.619 |
| ebi-a-GCST90017015 | Gut microbiota abundance (genus Holdemania id.2157) | 0.001 | 0.808 |
| ebi-a-GCST90017016 | Gut microbiota abundance (genus Howardella id.2000) | 0.003 | 0.425 |
| ebi-a-GCST90017017 | Gut microbiota abundance (genus Hungatella id.11306) | -0.005 | 0.220 |
| ebi-a-GCST90017018 | Gut microbiota abundance (genus Intestinibacter id.11345) | 0.005 | 0.112 |
| ebi-a-GCST90017019 | Gut microbiota abundance (genus Intestinimonas id.2062) | -0.001 | 0.806 |
| ebi-a-GCST90017020 | Gut microbiota abundance (genus Lachnoclostridium id.11308) | -0.002 | 0.536 |
| ebi-a-GCST90017021 | Gut microbiota abundance (genus Lachnospira id.2004) | -0.001 | 0.574 |
| ebi-a-GCST90017022 | Gut microbiota abundance (genus Lachnospiraceae FCS020 group id.11314) | 0.000 | 0.969 |
| ebi-a-GCST90017023 | Gut microbiota abundance (genus Lachnospiraceae NC2004 group id.11316) | 0.001 | 0.809 |
| ebi-a-GCST90017024 | Gut microbiota abundance (genus Lachnospiraceae ND3007 group id.11317) | -0.003 | 0.092 |
| ebi-a-GCST90017025 | Gut microbiota abundance (genus Lachnospiraceae NK4A136 group id.11319) | -0.001 | 0.593 |
| ebi-a-GCST90017026 | Gut microbiota abundance (genus Lachnospiraceae UCG001 id.11321) | 0.000 | 0.927 |
| ebi-a-GCST90017027 | Gut microbiota abundance (genus Lachnospiraceae UCG004 id.11324) | -0.004 | 0.180 |
| ebi-a-GCST90017028 | Gut microbiota abundance (genus Lachnospiraceae UCG008 id.11328) | -0.003 | 0.216 |
| ebi-a-GCST90017029 | Gut microbiota abundance (genus Lachnospiraceae UCG010 id.11330) | 0.010 | 0.522 |
| ebi-a-GCST90017030 | Gut microbiota abundance (genus Lactobacillus id.1837) | -0.004 | 0.205 |
| ebi-a-GCST90017031 | Gut microbiota abundance (genus Lactococcus id.1851) | 0.002 | 0.506 |
| ebi-a-GCST90017032 | Gut microbiota abundance (genus Marvinbryantia id.2005) | 0.001 | 0.596 |
| ebi-a-GCST90017033 | Gut microbiota abundance (genus Methanobrevibacter id.123) | -0.007 | 0.039 |
| ebi-a-GCST90017034 | Gut microbiota abundance (genus Odoribacter id.952) | 0.001 | 0.811 |
| ebi-a-GCST90017035 | Gut microbiota abundance (genus Olsenella id.822) | -0.008 | 0.036 |
| ebi-a-GCST90017036 | Gut microbiota abundance (genus Oscillibacter id.2063) | 0.005 | 0.085 |
| ebi-a-GCST90017037 | Gut microbiota abundance (genus Oscillospira id.2064) | 0.006 | 0.058 |
| ebi-a-GCST90017038 | Gut microbiota abundance (genus Oxalobacter id.2978) | -0.001 | 0.684 |
| ebi-a-GCST90017039 | Gut microbiota abundance (genus Parabacteroides id.954) | 0.001 | 0.613 |
| ebi-a-GCST90017040 | Gut microbiota abundance (genus Paraprevotella id.962) | 0.002 | 0.541 |
| ebi-a-GCST90017041 | Gut microbiota abundance (genus Parasutterella id.2892) | 0.002 | 0.443 |
| ebi-a-GCST90017042 | Gut microbiota abundance (genus Peptococcus id.2037) | -0.005 | 0.129 |
| ebi-a-GCST90017043 | Gut microbiota abundance (genus Phascolarctobacterium id.2168) | -0.002 | 0.629 |
| ebi-a-GCST90017044 | Gut microbiota abundance (genus Prevotella7 id.11182) | 0.001 | 0.863 |
| ebi-a-GCST90017045 | Gut microbiota abundance (genus Prevotella9 id.11183) | 0.002 | 0.436 |
| ebi-a-GCST90017046 | Gut microbiota abundance (genus Rikenellaceae RC9 gut group id.11191) | 0.000 | 0.928 |
| ebi-a-GCST90017047 | Gut microbiota abundance (genus Romboutsia id.11347) | -0.002 | 0.379 |
| ebi-a-GCST90017048 | Gut microbiota abundance (genus Roseburia id.2012) | 0.000 | 0.858 |
| ebi-a-GCST90017049 | Gut microbiota abundance (genus Ruminiclostridium5 id.11355) | -0.002 | 0.411 |
| ebi-a-GCST90017050 | Gut microbiota abundance (genus Ruminiclostridium6 id.11356) | 0.000 | 0.987 |
| ebi-a-GCST90017051 | Gut microbiota abundance (genus Ruminiclostridium9 id.11357) | 0.003 | 0.199 |
| ebi-a-GCST90017052 | Gut microbiota abundance (genus Ruminococcaceae NK4A214 group id.11358) | -0.003 | 0.305 |
| ebi-a-GCST90017053 | Gut microbiota abundance (genus Ruminococcaceae UCG002 id.11360) | -0.002 | 0.464 |
| ebi-a-GCST90017054 | Gut microbiota abundance (genus Ruminococcaceae UCG003 id.11361) | 0.000 | 0.940 |
| ebi-a-GCST90017055 | Gut microbiota abundance (genus Ruminococcaceae UCG004 id.11362) | -0.001 | 0.771 |
| ebi-a-GCST90017056 | Gut microbiota abundance (genus Ruminococcaceae UCG005 id.11363) | -0.002 | 0.390 |
| ebi-a-GCST90017057 | Gut microbiota abundance (genus Ruminococcaceae UCG009 id.11366) | -0.001 | 0.743 |
| ebi-a-GCST90017058 | Gut microbiota abundance (genus Ruminococcaceae UCG010 id.11367) | -0.004 | 0.143 |
| ebi-a-GCST90017059 | Gut microbiota abundance (genus Ruminococcaceae UCG011 id.11368) | 0.007 | 0.115 |
| ebi-a-GCST90017060 | Gut microbiota abundance (genus Ruminococcaceae UCG013 id.11370) | 0.001 | 0.543 |
| ebi-a-GCST90017061 | Gut microbiota abundance (genus Ruminococcaceae UCG014 id.11371) | 0.002 | 0.231 |
| ebi-a-GCST90017062 | Gut microbiota abundance (genus Ruminococcus gauvreauii group id.11342) | 0.002 | 0.455 |
| ebi-a-GCST90017063 | Gut microbiota abundance (genus Ruminococcus gnavus group id.14376) | -0.004 | 0.098 |
| ebi-a-GCST90017064 | Gut microbiota abundance (genus Ruminococcus torques group id.14377) | -0.003 | 0.363 |
| ebi-a-GCST90017065 | Gut microbiota abundance (genus Ruminococcus1 id.11373) | -0.003 | 0.248 |
| ebi-a-GCST90017066 | Gut microbiota abundance (genus Ruminococcus2 id.11374) | 0.000 | 0.949 |
| ebi-a-GCST90017067 | Gut microbiota abundance (genus Sellimonas id.14369) | -0.003 | 0.397 |
| ebi-a-GCST90017068 | Gut microbiota abundance (genus Senegalimassilia id.11160) | 0.000 | 0.919 |
| ebi-a-GCST90017069 | Gut microbiota abundance (genus Slackia id.825) | -0.002 | 0.644 |
| ebi-a-GCST90017070 | Gut microbiota abundance (genus Streptococcus id.1853) | -0.001 | 0.798 |
| ebi-a-GCST90017071 | Gut microbiota abundance (genus Subdoligranulum id.2070) | -0.001 | 0.772 |
| ebi-a-GCST90017072 | Gut microbiota abundance (genus Sutterella id.2896) | -0.001 | 0.803 |
| ebi-a-GCST90017073 | Gut microbiota abundance (genus Terrisporobacter id.11348) | 0.002 | 0.445 |
| ebi-a-GCST90017074 | Gut microbiota abundance (genus Turicibacter id.2162) | -0.003 | 0.205 |
| ebi-a-GCST90017075 | Gut microbiota abundance (genus Tyzzerella3 id.11335) | 0.003 | 0.405 |
| ebi-a-GCST90017076 | Gut microbiota abundance (genus Veillonella id.2198) | 0.002 | 0.347 |
| ebi-a-GCST90017077 | Gut microbiota abundance (unknown genus id.1000000073) | 0.003 | 0.256 |
| ebi-a-GCST90017078 | Gut microbiota abundance (unknown genus id.1000001215) | 0.001 | 0.566 |
| ebi-a-GCST90017079 | Gut microbiota abundance (unknown genus id.1000005472) | 0.008 | 0.012 |
| ebi-a-GCST90017080 | Gut microbiota abundance (unknown genus id.1000005479) | -0.001 | 0.743 |
| ebi-a-GCST90017081 | Gut microbiota abundance (unknown genus id.1000006162) | 0.005 | 0.118 |
| ebi-a-GCST90017082 | Gut microbiota abundance (unknown genus id.1868) | 0.000 | 0.872 |
| ebi-a-GCST90017083 | Gut microbiota abundance (unknown genus id.2001) | 0.000 | 0.953 |
| ebi-a-GCST90017084 | Gut microbiota abundance (unknown genus id.2041) | -0.001 | 0.657 |
| ebi-a-GCST90017085 | Gut microbiota abundance (unknown genus id.2071) | -0.002 | 0.448 |
| ebi-a-GCST90017086 | Gut microbiota abundance (unknown genus id.2755) | 0.001 | 0.765 |
| ebi-a-GCST90017087 | Gut microbiota abundance (unknown genus id.826) | 0.004 | 0.400 |
| ebi-a-GCST90017088 | Gut microbiota abundance (unknown genus id.959) | -0.003 | 0.164 |
